# Systemic inflammation indicators and risk of incident arrhythmias in 478,524 individuals: evidence from the UK Biobank cohort

**DOI:** 10.1186/s12916-023-02770-5

**Published:** 2023-02-28

**Authors:** Xiaorong Yang, Shaohua Zhao, Shaohua Wang, Xuelei Cao, Yue Xu, Meichen Yan, Mingmin Pang, Fan Yi, Hao Wang

**Affiliations:** 1 Clinical Epidemiology Unit, Qilu Hospital of Shandong University, Jinan, China; 2 Clinical Research Center of Shandong University, Qilu Hospital, Cheeloo College of Medicine, Shandong University, Jinan, China; 3 Department of Geriatric Medicine, Qilu Hospital of Shandong University, Jinan, China; 4 Key Laboratory of Cardiovascular Proteomics of Shandong Province, Qilu Hospital of Shandong University, Jinan, China; 5 Department of Internal Medicine, Jinan Hospital, Jinan, China; 6 Department of Clinical Laboratory, Qilu Hospital of Shandong University, Jinan, Shandong China; 7 Qilu Hospital of Shandong University, Jinan, China; 8 Department of Pharmacology, School of Basic Medical Sciences, Shandong University, Jinan, Shandong China; 9 Department of Critical Care Medicine, Qilu Hospital of Shandong University, Jinan, Shandong China

**Keywords:** Atrial fibrillation, Ventricular arrhythmia, Bradyarrhythmia, Systemic inflammation, Blood-based inflammation markers, UK Biobank

## Abstract

**Background:**

The role of systemic inflammation in promoting cardiovascular diseases has attracted attention, but its correlation with various arrhythmias remains to be clarified. We aimed to comprehensively assess the association between various indicators of systemic inflammation and atrial fibrillation/flutter (AF), ventricular arrhythmia (VA), and bradyarrhythmia in the UK Biobank cohort.

**Methods:**

After excluding ineligible participants, a total of 478,524 eligible individuals (46.75% male, aged 40–69 years) were enrolled in the study to assess the association between systemic inflammatory indicators and each type of arrhythmia.

**Results:**

After covariates were fully adjusted, CRP levels were found to have an essentially linear positive correlation with the risk of various arrhythmias; neutrophil count, monocyte count, and NLR showed a non-linear positive correlation; and lymphocyte count, SII, PLR, and LMR showed a U-shaped association. VA showed the strongest association with systemic inflammation indicators, and it was followed sequentially by AF and bradyarrhythmia.

**Conclusions:**

Multiple systemic inflammatory indicators showed strong associations with the onset of AF, VA, and bradyarrhythmia, of which the latter two have been rarely studied. Active systemic inflammation management might have favorable effects in reducing the arrhythmia burden and further randomized controlled studies are needed.

**Supplementary Information:**

The online version contains supplementary material available at 10.1186/s12916-023-02770-5.

## Background

Arrhythmias are a global challenge to human health [1]. The prevalence of arrhythmias is estimated to be 1.5% to 5% in the general population [2] and increases rapidly with age [1–4]. Atrial fibrillation/flutter (AF), ventricular arrhythmia (VA), and bradyarrhythmia are the most common types of arrhythmias that cause serious adverse outcomes [5, 6]. It is important to assess for risk factors to reduce the likelihood of arrhythmias onset in the first place.

Systemic inflammation, the result of the release of proinflammatory cytokines and chronic activation of the innate immune system, has been implicated in the development of some chronic diseases [7–11]. Although there is emerging evidence on the role of inflammatory dysregulation in AF [12–16], those studies usually used a single biomarker and showed inconsistent results. Furthermore, the relationship between inflammatory markers and VA/bradyarrhythmia is rarely reported. Large prospective studies that involve multitudinous inflammatory indicators and provide high-level evidence are needed to systematically explore the association between inflammation and different types of arrhythmias.

The UK Biobank (UKB) is a prospective cohort containing in-depth health information. Using this large-scale database, we systematically explored the relationship between eight systemic inflammation indicators and the incidence of AF, VA, and bradyarrhythmia.

## Methods

### Study population

The UKB enrolled more than 500,000 participants aged 40 to 69 years in 2006–2010 [17]. In brief, the clinical, genetic, and biochemical data of participants were obtained through questionnaires, genotyping, sample assays, physical measures, and linked electronic health data. Health outcomes were tracked for all the participants through linkage to national e-health-related datasets. Ethical approval for the UKB study was obtained from the National Information Governance Board for Health and Social Care and the National Health Service North West Multicenter Research Ethics Committee. Participants provided their written informed consent at baseline. This study utilized UKB resources under application number 82232 and followed the Strengthening the Reporting of Observational Studies in Epidemiology (STROBE) reporting guidelines.

**Fig. 1:**
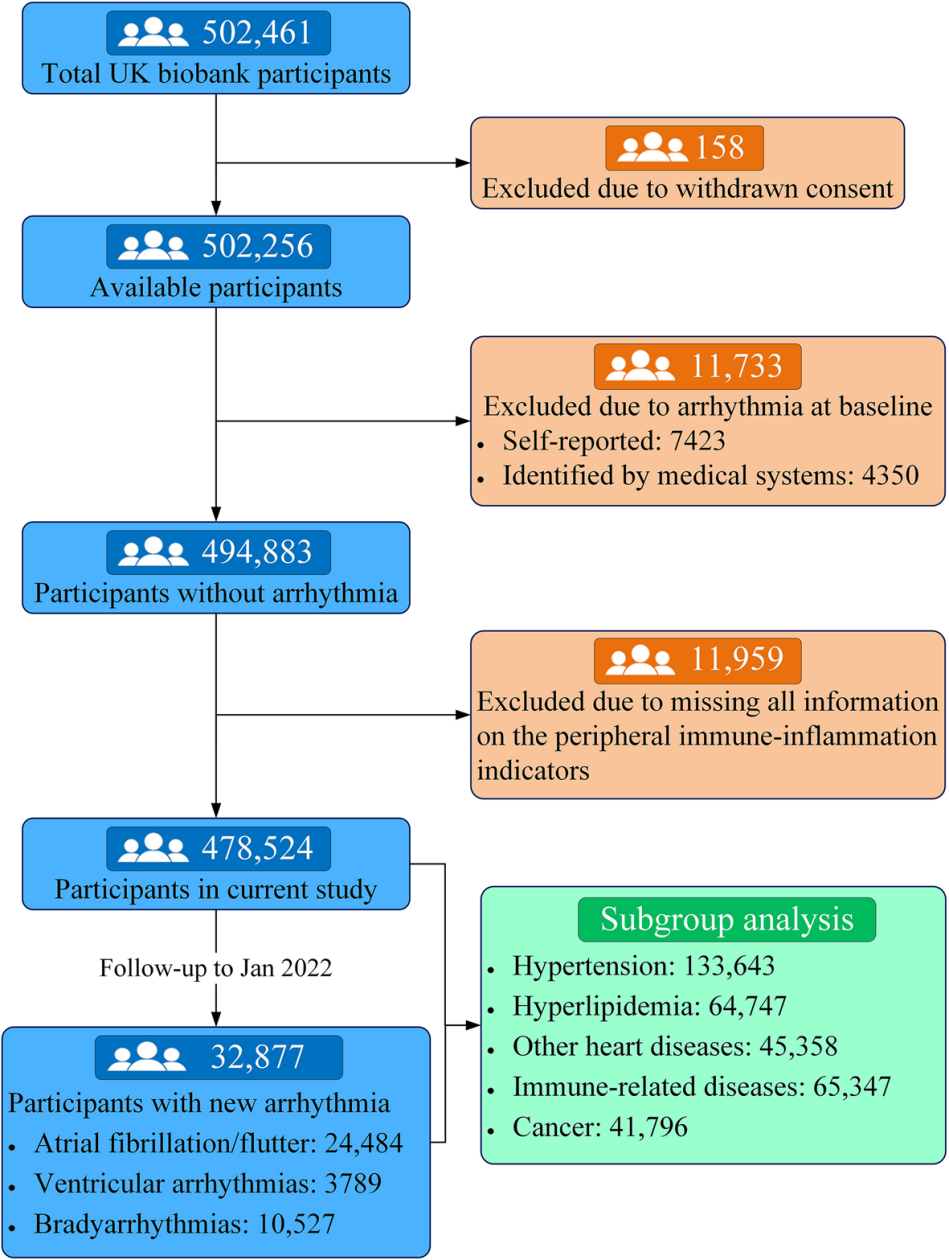
The flow diagram of the UK biobank participants in this study

In the current analysis, we excluded participants who subsequently withdrew their consent (*n* = 158); those with AF, VA, or bradyarrhythmia at baseline (*n* = 11,773); and those for whom information on systemic inflammatory markers was missing (*n* = 11,959). As a result, the primary analysis included a final group of 478,524 participants (Fig. 1).

### Systemic inflammation indicators

The quality check procedure for blood sample data carried out at UKB is available at https://biobank.ndph.ox.ac.uk/showcase/showcase/docs/biomarker_issues.pdf. The instrument reports 31 parameters, the details of which are available at https://biobank.ndph.ox.ac.uk/showcase/ukb/docs/haematology.pdf. We extracted baseline count data for neutrophils, monocytes, lymphocytes, and platelets. Based on the blood cells counts, we calculated the values of combined inflammation indicators, including the neutrophil-to-lymphocyte ratio (NLR, neutrophils/lymphocytes), lymphocyte-to-monocyte ratio (LMR, lymphocytes/monocytes), platelet-to-lymphocyte ratio (PLR, platelets/lymphocytes), and systemic immune-inflammation index (SII, neutrophils × platelets/lymphocytes), which have been demonstrated to predict inflammatory status under several conditions in previous studies [18–20]. Moreover, serum C-reactive protein (CRP) was included in the current study and was detected by immunoturbidimetric high-sensitivity assays on a Beckman Coulter AU5800.

### Outcome ascertainment

The primary outcomes of interest were AF, VA, and bradyarrhythmia, according to follow-up data obtained up to January 2022. Incident AF, VA, and bradyarrhythmia were identified based on the International Classification of Diseases, Ninth Revision (ICD-9) and International Statistical Classification of Diseases and Related Health Problems, Tenth Revision (ICD-10) codes available from inpatient and outpatient records and causes of death for a linked medical encounter. We also used the occurrence of a relevant operative procedure for each arrhythmias subtype. Detailed disease definitions are provided in Additional file 1: Table S1.

### Assessment of covariates

Race and ethnicity were ascertained as basic demographic variables and classified according to self-reports from the interviews. For the education levels, the College or University degree was described as College degree; the A levels/AS levels or equivalent was described as High school graduate; the O levels/GCSEs or equivalent was described as Middle school graduate, and other types were described as None of the above. The Townsend deprivation index was obtained based on participants’ zip codes: higher values of the index indicated higher levels of deprivation. Body mass index (BMI) was derived from measurements of height and weight obtained during the initial assessment and was calculated by dividing weight (kilograms) by height (meters) squared. Physical activity was categorized into tertiles according to participants’ responses to the question on the number of days of moderate physical activity for over 10 min per week. Participants were classified as non-drinkers, light-to-moderate drinkers, or heavy drinkers based on their self-reported average daily alcohol consumption.

The presence of hypertension, myocardial infarction, angina, stroke, diabetes, hyperlipidemia, diseases of blood and blood-forming organs (DBBF), chronic diseases involving the immune mechanism, and malignant neoplasms at baseline was determined based on self-reported diseases and the ICD-9 and ICD-10 codes from inpatient and/or outpatient visits before the date of attending the assessment center. The definitions of these diseases are provided in Additional file 1: Table S2.

### Statistical analysis

Continuous variables are represented by mean and standard deviation, and categorical variables are represented by proportion. We used Cox proportional hazard ratio (HR) and 95% confidence interval (CI) to assess the association between systemic inflammatory indicators and each type of arrhythmia. Three major models were fitted: model 1 included age (continuous variable), sex, and race as covariates; model 2 included additional potential confounders, such as education, Townsend deprivation index, smoking status, frequency of alcohol drinking, BMI, and physical activity; and model 3 further included related diseases at baseline, including hypertension, myocardial infarction, angina, stroke, diabetes, hyperlipidemia, DBBF, chronic diseases involving the immune mechanism, and malignant neoplasms (Table 1). With systemic inflammation indicators as a continuous exposure variable, we used restricted cubic splines with 5 knots placed at the 5th, 27.5th, 50th, 72.5th, and 95th percentiles to assess the potential non-linear effect of systemic inflammation status on arrhythmias, after shrinking 1‰ outliers of systemic inflammation indicators. To easily compare the associations of various systemic inflammation indicators with the three arrhythmia subtypes for different models and conditions, the systemic inflammation indicators were further divided into seven categories, taking into account the normal reference range, data distribution, and easy-to-understand numbers. The following sensitivity analyses were performed: (1) Participants with events in the first 2 years of follow-up were excluded to mitigate any potential effects of reverse causality. (2) Participants with malignant neoplasms, DBBF, heart diseases, and chronic diseases involving the immune mechanism at baseline were excluded to mitigate any potential bias due to survivorship. All analyses were conducted using STATA version 15.1 (Stata Corporation, College Station, TX, USA). Two-tailed *p*-values less than 0.05 were considered to indicate significance.

**Table 1:** Demographic and clinical characteristics of participants in a study of arrhythmias in the UK Biobank

Characteristic	Total ***N***=478,524	None of arrhythmias ***N***=445,647	Any arrhythmias ***N***=32,877	Atrial fibrillation/flutter ***N***=24,484	Ventricular arrhythmia ***N***=3789	Bradyarrhythmia ***N***=10,527
**Age (years)**	56.4 ± 8.1	56.0 ± 8.1	61.7 ± 6.3	62.0 ± 6.0	60.3 ± 7.1	61.8 ± 6.3
**Female,** ***n*** **(%)**	261,564 (54.66)	249,713 (56.03)	11,851 (36.05)	9139 (37.33)	1155 (30.48)	3237 (30.75)
**White race,** ***n*** **(%)**	450,543 (94.60)	418,996 (94.46)	31,547 (96.53)	23,701 (97.34)	3544 (94.13)	9974 (95.44)
**Townsend deprivation index**	−1.3 ± 3.1	−1.3 ± 3.1	−1.2 ± 3.2	−1.2 ± 3.2	−0.8 ± 3.3	−1.2 ± 3.1
**Educational level,** ***n*** **(%)**						
College degree	155,367 (32.86)	147,043 (33.38)	8324 (25.70)	6113 (25.34)	940 (25.26)	2727 (26.29)
High school graduate	53,340 (11.28)	50,480 (11.46)	2860 (8.83)	2119 (8.78)	349 (9.38)	863 (8.32)
Middle school graduate	101,327 (21.43)	94,899 (21.55)	6428 (19.85)	4817 (19.97)	726 (19.51)	2049 (19.76)
None of the above	162,808 (34.43)	148,033 (33.61)	14,775 (45.62)	11,074 (45.91)	1707 (45.86)	4732 (45.86)
**Body mass index (kg/m**^**2**^**)**	27.39 ± 4.8	27.3 ± 4.7	28.9 ± 5.3	29.1 ± 5.4	28.6 ± 5.2	28.8 ± 5.0
**Smoking status,** ***n*** **(%)**						
Never	261,551 (54.94)	246,970 (55.69)	14,581 (44.66)	10,736 (44.17)	1539 (40.90)	4835 (46.27)
Former	164,013 (34.45)	149,806 (33.78)	14,207 (43.52)	10,768 (44.30)	1592 (42.31)	4544 (43.49)
Current	50,525 (10.61)	46,665 (10.52)	3860 (11.82)	2803 (11.53)	632 (16.80)	1070 (10.24)
**Daily alcohol,** ***n*** **(%)**						
Daily or almost daily	96,967 (20.31)	89,261 (20.07)	7706 (23.51)	5903 (24.18)	857 (22.68)	2357 (22.46)
Three or four times a week	110,464 (23.14)	103,313 (23.23)	7151 (21.82)	5318 (21.79)	807 (21.36)	2295 (21.87)
Once or twice a week	123,611 (25.89)	115,826 (26.05)	7785 (23.75)	5832 (23.89)	897 (23.74)	2531 (24.11)
Less once a week	108,252 (22.67)	101,181 (22.75)	7071 (21.57)	5153 (21.11)	820 (21.70)	2315 (22.06)
Never	38,175 (8.00)	35,108 (7.89)	3067 (9.36)	2204 (9.03)	397 (10.51)	998 (9.51)
**Physical activity,** ***n*** **(%)**						
Light	160,870 (35.49)	150,391 (35.59)	10,479 (34.21)	7780 (34.14)	1249 (35.88)	3280 (33.35)
Moderate	113,373 (25.01)	105,933 (25.07)	7440 (24.29)	5576 (24.47)	831 (23.87)	2429 (24.70)
High	178,998 (39.49)	166,290 (39.35)	12,708 (41.49)	9430 (41.39)	1401 (40.25)	4125 (41.95)
**Disease history at baseline**						
Hypertension	133,643 (27.93)	117,606 (26.39)	16,037 (48.78)	12,162 (49.68)	1824 (48.14)	5217 (49.56)
Heart attack	10,038 (2.10)	7597 (1.70)	2441 (7.43)	1692 (6.91)	488 (12.88)	916 (8.70)
Angina	17,719 (3.70)	14,001 (3.14)	3718 (11.31)	2681 (10.95)	533 (14.07)	1388 (13.19)
Stroke	7360 (1.54)	6071 (1.36)	1289 (3.92)	1001 (4.09)	146 (3.85)	407 (3.87)
Diabetes	25,316 (5.29)	21,439 (4.81)	3877 (11.79)	2772 (11.32)	548 (14.46)	1453 (13.80)
Hyperlipidemia	64,747 (13.53)	56,398 (12.66)	8349 (25.40)	6120 (25.00)	1042 (27.51)	2966 (28.19)
Diseases of blood and blood-forming organs (DBBF)	4320 (0.90)	3846 (0.86)	474 (1.44)	375 (1.53)	74 (1.95)	109 (1.04)
Chronic diseases involving the immune mechanism	65,347 (13.66)	60,361 (13.55)	4986 (15.17)	3749 (15.32)	618 (16.31)	1526 (14.50)
Malignant neoplasms	41,796 (8.74)	37,867 (8.50)	3929 (11.95)	3046 (12.44)	423 (11.17)	1135 (10.79)
**Systemic inflammation indicators**						
C-reactive protein (CRP, mg/L)	2.59 ± 4.34	2.53 ± 4.25	3.30 ± 5.32	3.36 ± 5.37	3.58 ± 6.02	3.07 ± 4.80
Neutrophil count (10^9 cells/L)	4.22 ± 1.42	4.20 ± 1.41	4.49 ± 1.52	4.49 ± 1.52	4.67 ± 1.66	4.45 ± 1.48
Monocyte count (10^9 cells/L)	0.47 ± 0.27	0.47 ± 0.27	0.52 ± 0.30	0.52 ± 0.33	0.54 ± 0.22	0.52 ± 0.22
Lymphocyte count (10^9 cells/L)	1.97 ± 1.15	1.97 ± 1.15	1.96 ± 1.19	1.96 ± 1.27	1.98 ± 1.04	1.97 ± 1.01
Systemic immune-inflammation index (SII)	598.99 ± 367.44	596.47 ± 355.89	633.07 ± 496.89	635.53 ± 525.87	673.37 ± 762.95	616.75 ± 391.01
Neutrophil-to-lymphocyte ratio (NLR)	2.35 ± 1.24	2.34 ± 1.20	2.57 ± 1.76	2.59 ± 1.88	2.71 ± 2.90	2.53 ± 1.32
Platelet-to-lymphocyte ratio (PLR)	142.07 ± 69.11	142.23 ± 67.88	139.96 ± 84.00	140.18 ± 90.12	142.69 ± 148.84	137.89 ± 60.54
Lymphocyte-to-monocyte ratio (LMR)	4.64 ± 4.26	4.67 ± 4.32	4.23 ± 3.17	4.21 ± 3.12	4.22 ± 4.04	4.21 ± 2.86
**Follow-up years (years)**	12.21 ± 2.35	12.58 ± 1.75	7.17 ± 3.32	7.13 ± 3.36	7.24 ± 3.28	7.79 ± 3.06

## Results

### Population characteristics

The baseline characteristics and systemic inflammatory indicators of participants (478,524) are presented according to the presence of arrhythmias and the subtypes (Table 1). In general, the mean age of all the participants was 56.4 ± 8.1 years, and 261,564 (46.75%) were male. Over a mean follow-up period of 12.2 years, 32,877 participants developed arrhythmias, including 24,484 cases of AF, 3789 cases of VA, and 10,527 cases of bradyarrhythmia.

Compared with the 445,647 participants in the control group, the 32,877 participants in the incident arrhythmia group were more likely to be older, male, and less educated, and tended to have higher BMI, higher systolic blood pressure, a higher smoking rate, and a higher prevalence of comorbid diseases (Table 1). Considering the possible correlation between systemic inflammation levels and baseline characteristics, we have shown the baseline characteristics of patients for different levels of CRP (as an indicator of systemic inflammation). Participants with higher CRP levels were more likely to be older, less educated, and smokers, and tended to have a higher Townsend deprivation index, higher BMI, higher systolic and diastolic blood pressure, lower alcohol consumption, lower physical activity intensity, and higher prevalence of comorbid diseases (Additional file 1: Table S3).

### Atrial fibrillation/flutter

**Fig. 2:**
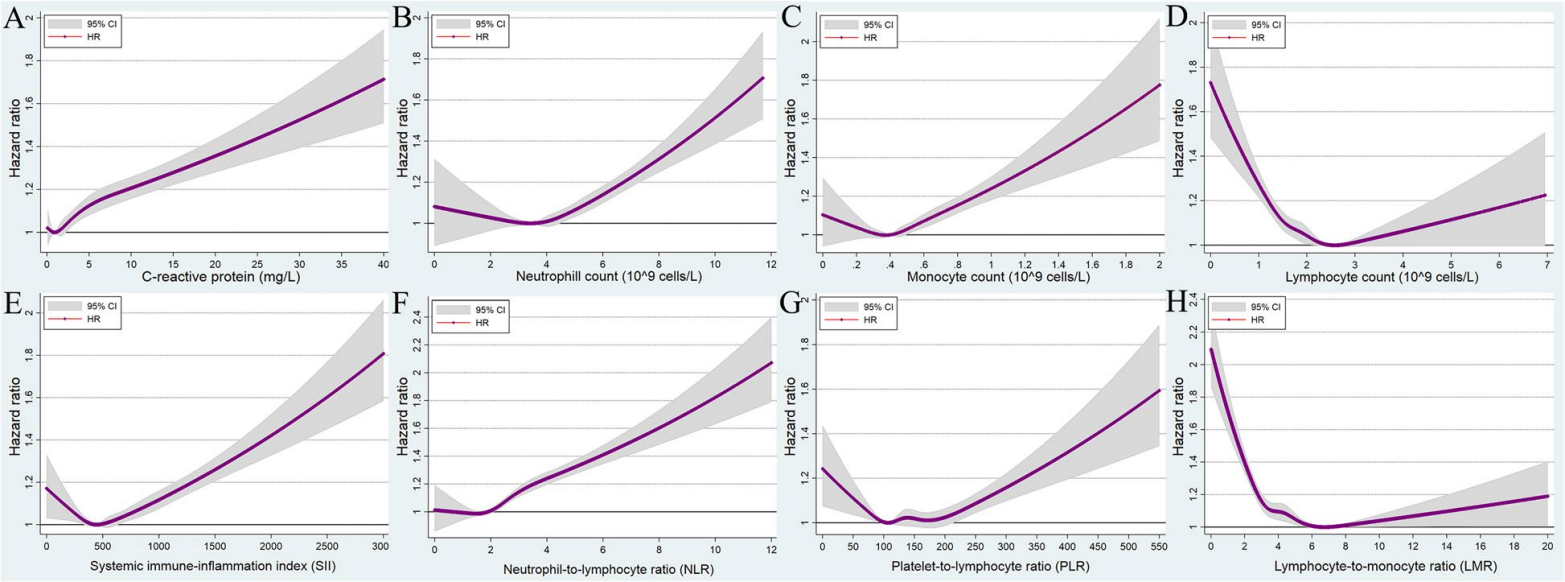
Multivariable-adjusted association between different systematic information indicators and the risk of atrial fibrillation/flutter by restricted cubic spline regression. **A** C-reactive protein; **B** neutrophil count; **C** monocyte count; **D** lymphocyte count; **E** systemic immune-inflammation index (neutrophils × platelets/lymphocytes); **F** neutrophil-to-lymphocyte ratio (neutrophils/lymphocytes); **G** platelet-to-lymphocyte ratio (platelets/lymphocytes); **H** lymphocyte-to-monocyte ratio (lymphocytes/monocytes). HR, hazard ratio; CI, confidence intervals

There were 24,484 incident AF events across 5.88 million person-years of follow-up (incidence rate: 4.16 events per 1000 person-years, 95% CI: 4.11–4.21). After adjusting for all potential confounding variables, the CRP levels were significantly and positively associated with the risk of incident AF (Fig. 2A). Compared with the reference population with a CRP of <0.5 mg/L, the risk of incident AF in the population with CRP >10 mg/L was 1.33 (95% CI: 1.24–1.43) (Table 2). Moreover, we found that the HR for incident AF increased significantly with an increase in the neutrophil count, monocyte count, and NLR, although a slight opposite trend (not statistically significant) was found at the low neutrophil count, monocyte count, and NLR, (Fig. 2B, C, and F; Table 2).

**Table 2:** The association between various systematic information indicators and three arrhythmia subtypes

Variables	Atrial fibrillation/flutter			Ventricular arrhythmia			Bradyarrhythmia		
	Model 1	Model 2	Model 3	Model 1	Model 2	Model 3	Model 1	Model 2	Model 3
**C-reactive protein (mg/L)**									
<0.5	1.00 (Ref.)	1.00 (Ref.)	1.00 (Ref.)	1.00 (Ref.)	1.00 (Ref.)	1.00 (Ref.)	1.00 (Ref.)	1.00 (Ref.)	1.00 (Ref.)
(0.5, 1.0)	1.10 (1.05~1.16)	0.99 (0.94~1.04)	1.00 (0.95~1.05)	1.10 (0.97~1.25)	1.03 (0.90~1.17)	1.06 (0.93~1.20)	1.11 (1.04~1.20)	1.02 (0.94~1.09)	1.04 (0.97~1.13)
(1.0, 2.0)	1.21 (1.16~1.27)	0.98 (0.94~1.03)	1.00 (0.95~1.05)	1.32 (1.17~1.48)	1.15 (1.01~1.30)	1.20 (1.06~1.36)	1.16 ((1.08~1.24)	0.98 (0.91~1.06)	1.03 (0.96~1.11)
(2.0, 3.0)	1.37 (1.30~1.44)	1.02 (0.96~1.08)	1.04 (0.98~1.10)	1.32 (1.16~1.51)	1.06 (0.92~1.23)	1.13 (0.98~1.30)	1.26 (1.17~1.37)	1.01 (0.93~1.10)	1.07 (0.98~1.16)
(3.0, 4.0)	1.47 (1.39~1.56)	1.04 (0.97~1.10)	1.06 (0.99~1.13)	1.54 (1.32~1.79)	1.20 (1.02~1.42)	1.28 (1.09~1.51)	1.42 (1.30~1.55)	1.08 (0.98~1.19)	1.15 (1.05~1.27)
(4.0, 10.0)	1.72 (1.63~1.81)	1.12 (1.06~1.19)	1.14 (1.07~1.20)	1.92 (1.69~2.18)	1.37 (1.19~1.58)	1.45 (1.26~1.67)	1.54 (1.42~1.66)	1.11 (1.02~1.21)	1.18 (1.08~1.29)
>=10.0	2.10 (1.97~2.23)	1.33 (1.24~1.43)	1.33 (1.24~1.43)	2.55 (2.18~2.98)	1.81 (1.53~2.16)	1.87 (1.57~2.22)	1.68 (1.52~1.86)	1.23 (1.10~1.37)	1.30 (1.16~1.45)
**Neutrophil count (10^9 cells/L)**									
<2.0	1.00 (Ref.)	1.00 (Ref.)	1.00 (Ref.)	1.00 (Ref.)	1.00 (Ref.)	1.00 (Ref.)	1.00 (Ref.)	1.00 (Ref.)	1.00 (Ref.)
(2.0, 3.0)	0.97 (0.86~1.09)	1.00 (0.88~1.13)	1.02 (0.90~1.16)	1.06 (0.77~1.45)	1.07 (0.77~1.49)	1.10 (0.79~1.53)	0.85 (0.72~1.00)	0.84 (0.70~1.00)	0.85 (0.71~1.01)
(3.0, 4.0)	1.02 (0.91~1.14)	0.98 (0.87~1.11)	0.99 (0.88~1.11)	1.21 (0.90~1.64)	1.13 (0.82~1.56)	1.14 (0.83~1.56)	0.93 (0.80~1.10)	0.89 (0.75~1.05)	0.88 (0.74~1.04)
(4.0, 5.0)	1.14 (1.02~1.28)	1.05 (0.93~1.18)	1.03 (0.91~1.16)	1.34 (0.99~1.81)	1.19 (0.87~1.64)	1.17 (0.85~1.60)	1.02 (0.87~1.20)	0.93 (0.79~1.10)	0.90 (0.76~1.06)
(5.0, 6.0)	1.29 (1.15~1.45)	1.12 (0.99~1.26)	1.08 (0.95~1.22)	1.82 (1.34~2.47)	1.52 (1.10~2.09)	1.43 (1.04~1.97)	1.15 (0.98~1.36)	1.01 (0.85~1.20)	0.94 (0.79~1.11)
(6.0, 7.5)	1.53 (1.36~1.72)	1.25 (1.10~1.41)	1.17 (1.03~1.33)	2.18 (1.59~2.97)	1.73 (1.24~2.40)	1.57 (1.13~2.18)	1.28 (1.08~1.51)	1.07 (0.90~1.28)	0.98 (0.82~1.17)
>=7.5	1.88 (1.65~2.13)	1.49 (1.30~1.71)	1.36 (1.19~1.55)	3.35 (2.42~4.64)	2.43 (1.72~3.43)	2.13 (1.51~3.01)	1.42 (1.17~1.71)	1.19 (0.98~1.45)	1.05 (0.86~1.28)
**Monocyte count (10^9 cells/L)**									
<0.3	1.00 (Ref.)	1.00 (Ref.)	1.00 (Ref.)	1.00 (Ref.)	1.00 (Ref.)	1.00 (Ref.)	1.00 (Ref.)	1.00 (Ref.)	1.00 (Ref.)
(0.3, 0.4)	0.98 (0.92~1.04)	1.00 (0.94~1.06)	1.00 (0.94~1.06)	0.91 (0.78~1.07)	0.93 (0.79~1.09)	0.93 (0.79~1.1)	0.95 (0.86~1.04)	0.97 (0.88~1.07)	0.97 (0.88~1.07)
(0.4, 0.5)	1.03 (0.98~1.09)	1.01 (0.95~1.07)	1.00 (0.94~1.06)	1.01 (0.87~1.17)	1.03 (0.88~1.20)	1.02 (0.87~1.19)	0.98 (0.90~1.07)	0.98 (0.90~1.08)	0.97 (0.89~1.06)
(0.5, 0.6)	1.11 (1.05~1.18)	1.04 (0.98~1.10)	1.02 (0.96~1.08)	1.17 (1.01~1.36)	1.11 (0.95~1.30)	1.09 (0.93~1.27)	1.06 (0.97~1.16)	1.01 (0.92~1.11)	0.99 (0.90~1.08)
(0.6, 0.7)	1.24 (1.17~1.32)	1.11 (1.04~1.19)	1.08 (1.01~1.15)	1.24 (1.06~1.45)	1.11 (0.94~1.31)	1.06 (0.90~1.25)	1.15 (1.05~1.26)	1.07 (0.97~1.18)	1.02 (0.93~1.13)
(0.7, 0.8)	1.40 (1.31~1.50)	1.20 (1.11~1.28)	1.13 (1.05~1.22)	1.57 (1.33~1.86)	1.38 (1.15~1.65)	1.27 (1.06~1.53)	1.34 (1.21~1.48)	1.19 (1.07~1.32)	1.11 (1.00~1.24)
>=0.8	1.58 (1.47~1.69)	1.28 (1.19~1.38)	1.18 (1.10~1.27)	2.14 (1.81~2.52)	1.79 (1.50~2.13)	1.58 (1.33~1.89)	1.3 (1.17~1.45)	1.14 (1.02~1.28)	1.03 (0.92~1.15)
**Lymphocyte count (10^9 cells/L)**									
<0.8	1.55 (1.37~1.75)	1.74 (1.53~1.98)	1.56 (1.38~1.78)	1.57 (1.17~2.12)	1.59 (1.14~2.22)	1.39 (0.99~1.95)	1.34 (1.11~1.63)	1.42 (1.15~1.74)	1.33 (1.08~1.64)
(0.8, 1.5)	1.01 (0.98~1.05)	1.15 (1.10~1.19)	1.13 (1.09~1.18)	1.04 (0.94~1.14)	1.18 (1.07~1.31)	1.16 (1.05~1.29)	0.97 (0.91~1.02)	1.04 (0.98~1.11)	1.04 (0.98~1.10)
(1.5, 2.0)	0.99 (0.96~1.03)	1.06 (1.02~1.1)	1.05 (1.02~1.09)	0.89 (0.82~0.98)	0.97 (0.88~1.06)	0.97 (0.88~1.06)	0.99 (0.94~1.05)	1.04 (0.98~1.09)	1.04 (0.98~1.09)
(2.0, 2.5)	1.00 (Ref.)	1.00 (Ref.)	1.00 (Ref.)	1.00 (Ref.)	1.00 (Ref.)	1.00 (Ref.)	1.00 (Ref.)	1.00 (Ref.)	1.00 (Ref.)
(2.5, 3.0)	1.04 (0.99~1.09)	0.98 (0.93~1.03)	0.97 (0.92~1.02)	1.09 (0.97~1.23)	1.01 (0.90~1.15)	0.99 (0.88~1.12)	1.02 (0.95~1.10)	0.96 (0.89~1.03)	0.94 (0.87~1.01)
(3.0, 4.0)	1.17 (1.10~1.24)	1.02 (0.95~1.08)	0.99 (0.93~1.06)	1.32 (1.14~1.53)	1.13 (0.97~1.32)	1.08 (0.92~1.26)	1.32 (1.21~1.44)	1.23 (1.12~1.35)	1.18 (1.07~1.29)
>=4.0	1.35 (1.18~1.53)	1.17 (1.02~1.35)	1.10 (0.96~1.27)	1.66 (1.23~2.25)	1.39 (1.00~1.93)	1.28 (0.92~1.77)	1.32 (1.08~1.61)	1.17 (0.94~1.46)	1.11 (0.89~1.38)
**Systemic immune-inflammation index (SII)**									
<300	1.08 (1.02~1.13)	1.07 (1.01~1.13)	1.07 (1.01~1.13)	1.15 (1.00~1.31)	1.15 (1.00~1.32)	1.14 (0.99~1.31)	1.11 (1.03~1.20)	1.10 (1.02~1.19)	1.11 (1.02~1.20)
(300, 400)	1.06 (1.01~1.10)	1.05 (1.00~1.10)	1.06 (1.01~1.11)	1.13 (1.01~1.28)	1.13 (1.00~1.27)	1.13 (1.00~1.28)	1.01 (0.94~1.08)	1.00 (0.93~1.07)	1.00 (0.93~1.08)
(400, 500)	1.00 (Ref.)	1.00 (Ref.)	1.00 (Ref.)	1.00 (Ref.)	1.00 (Ref.)	1.00 (Ref.)	1.00 (Ref.)	1.00 (Ref.)	1.00 (Ref.)
(500, 600)	1.05 (1.01~1.10)	1.04 (0.99~1.08)	1.03 (0.98~1.08)	1.16 (1.03~1.31)	1.14 (1.01~1.29)	1.14 (1.01~1.29)	1.03 (0.96~1.10)	1.02 (0.95~1.10)	1.02 (0.95~1.09)
(600, 800)	1.11 (1.06~1.16)	1.09 (1.04~1.13)	1.07 (1.02~1.11)	1.20 (1.07~1.34)	1.18 (1.05~1.32)	1.16 (1.03~1.30)	1.08 (1.01~1.15)	1.06 (1,00~1.14)	1.05 (0.98~1.12)
(800, 1500)	1.22 (1.17~1.27)	1.18 (1.13~1.23)	1.13 (1.08~1.19)	1.54 (1.38~1.72)	1.46 (1.30~1.65)	1.41 (1.25~1.58)	1.15 (1.08~1.23)	1.12 (1.04~1.20)	1.08 (1.01~1.16)
>=1500	1.69 (1.56~1.83)	1.62 (1.49~1.77)	1.48 (1.36~1.62)	2.88 (2.41~3.44)	2.65 (2.19~3.21)	2.39 (1.98~2.9)	1.39 (1.22~1.58)	1.37 (1.19~1.58)	1.29 (1.12~1.49)
**Neutrophil-to-lymphocyte ratio (NLR)**									
<1.5	1.00 (Ref.)	1.00 (Ref.)	1.00 (Ref.)	1.00 (Ref.)	1.00 (Ref.)	1.00 (Ref.)	1.00 (Ref.)	1.00 (Ref.)	1.00 (Ref.)
(1.5, 2.0)	1.00 (0.96~1.04)	1.00 (0.96~1.05)	0.99 (0.95~1.04)	1.04 (0.93~1.17)	1.02 (0.91~1.15)	1.01 (0.90~1.14)	0.99 (0.93~1.06)	0.99 (0.92~1.06)	0.98 (0.91~1.05)
(2.0, 2.5)	1.07 (1.02~1.12)	1.06 (1.02~1.11)	1.04 (1.00~1.09)	1.14 (1.02~1.28)	1.13 (1.00~1.27)	1.10 (0.98~1.24)	1.04 (0.97~1.11)	1.03 (0.97~1.11)	1.01 (0.94~1.08)
(2.5, 3.0)	1.15 (1.10~1.21)	1.13 (1.07~1.19)	1.10 (1.04~1.15)	1.18 (1.04~1.33)	1.14 (1.00~1.30)	1.10 (0.96~1.25)	1.06 (0.99~1.14)	1.05 (0.98~1.14)	1.02 (0.94~1.10)
(3.0, 3.5)	1.22 (1.16~1.29)	1.21 (1.14~1.28)	1.16 (1.10~1.22)	1.33 (1.16~1.53)	1.29 (1.12~1.49)	1.22 (1.06~1.41)	1.18 (1.09~1.28)	1.17 (1.08~1.28)	1.12 (1.03~1.21)
(3.5, 5.0)	1.36 (1.29~1.44)	1.36 (1.29~1.43)	1.28 (1.21~1.35)	1.69 (1.48~1.92)	1.61 (1.41~1.85)	1.49 (1.30~1.71)	1.19 (1.10~1.29)	1.17 (1.08~1.27)	1.10 (1.01~1.20)
>=5.0	1.64 (1.53~1.77)	1.62 (1.50~1.75)	1.46 (1.35~1.58)	2.33 (1.97~2.75)	2.18 (1.82~2.61)	1.91 (1.59~2.28)	1.38 (1.23~1.55)	1.36 (1.20~1.53)	1.23 (1.09~1.39)
**Platelet-to-lymphocyte ratio (PLR)**									
<80	1.22 (1.16~1.28)	1.11 (1.05~1.17)	1.08 (1.02~1.14)	1.40 (1.23~1.59)	1.22 (1.07~1.40)	1.15 (1.00~1.32)	1.23 (1.14~1.33)	1.14 (1.05~1.24)	1.10 (1.01~1.19)
(80, 100)	1.09 (1.05~1.14)	1.05 (1.00~1.10)	1.04 (0.99~1.09)	1.22 (1.08~1.36)	1.12 (1.00~1.27)	1.10 (0.97~1.24)	1.05 (0.98~1.13)	1.02 (0.95~1.09)	1.01 (0.94~1.08)
(100, 120)	1.00 (Ref.)	1.00 (Ref.)	1.00 (Ref.)	1.00 (Ref.)	1.00 (Ref.)	1.00 (Ref.)	1.00 (Ref.)	1.00 (Ref.)	1.00 (Ref.)
(120, 150)	0.99 (0.95~1.03)	1.02 (0.98~1.06)	1.02 (0.97~1.06)	1.01 (0.91~1.12)	1.04 (0.93~1.16)	1.04 (0.94~1.16)	0.97 (0.91~1.03)	0.98 (0.92~1.04)	0.98 (0.92~1.05)
(150, 200)	0.98 (0.94~1.02)	1.04 (0.99~1.08)	1.04 (0.99~1.08)	0.94 (0.85~1.05)	1.00 (0.9~1.12)	1.01 (0.90~1.13)	0.93 (0.87~0.99)	0.97 (0.91~1.03)	0.98 (0.92~1.04)
(200, 250)	0.98 (0.92~1.03)	1.06 (0.99~1.12)	1.04 (0.98~1.11)	1.17 (1.02~1.35)	1.24 (1.07~1.44)	1.24 (1.07~1.44)	0.96 (0.88~1.05)	1.01 (0.92~1.10)	1.01 (0.93~1.11)
>=250	1.12 (1.05~1.21)	1.26 (1.17~1.35)	1.19 (1.10~1.28)	1.50 (1.27~1.77)	1.56 (1.31~1.86)	1.47 (1.24~1.76)	1.06 (0.95~1.18)	1.14 (1.01~1.27)	1.11 (0.99~1.24)
**Lymphocyte-to-monocyte ratio (LMR)**									
<2.5	1.47 (1.39~1.56)	1.49 (1.40~1.58)	1.39 (1.30~1.47)	1.79 (1.54~2.07)	1.82 (1.55~2.13)	1.66 (1.42~1.95)	1.25 (1.15~1.37)	1.23 (1.12~1.35)	1.16 (1.06~1.27)
(2.5, 3.0)	1.23 (1.16~1.31)	1.24 (1.17~1.32)	1.19 (1.12~1.27)	1.54 (1.32~1.79)	1.63 (1.39~1.91)	1.55 (1.33~1.82)	1.06 (0.97~1.15)	1.04 (0.94~1.14)	1.00 (0.91~1.10)
(3.0, 4.0)	1.14 (1.08~1.20)	1.15 (1.09~1.21)	1.12 (1.06~1.19)	1.19 (1.04~1.37)	1.27 (1.10~1.47)	1.24 (1.07~1.43)	1.05 (0.98~1.14)	1.05 (0.97~1.14)	1.03 (0.95~1.11)
(4.0, 5.0)	1.07 (1.01~1.12)	1.08 (1.02~1.14)	1.07 (1.01~1.13)	1.15 (1.00~1.33)	1.2 (1.03~1.39)	1.19 (1.03~1.38)	0.99 (0.91~1.07)	0.98 (0.90~1.06)	0.97 (0.89~1.06)
(5.0, 6.0)	1.04 (0.98~1.10)	1.05 (0.99~1.11)	1.04 (0.98~1.11)	1.05 (0.90~1.22)	1.07 (0.91~1.26)	1.07 (0.91~1.27)	0.98 (0.89~1.07)	0.96 (0.87~1.05)	0.96 (0.87~1.05)
(6.0, 8.0)	1.00 (Ref.)	1.00 (Ref.)	1.00 (Ref.)	1.00 (Ref.)	1.00 (Ref.)	1.00 (Ref.)	1.00 (Ref.)	1.00 (Ref.)	1.00 (Ref.)
>=8.0	1.05 (0.97~1.14)	1.02 (0.94~1.11)	1.02 (0.93~1.11)	1.32 (1.08~1.61)	1.28 (1.03~1.58)	1.28 (1.03~1.58)	1.08 (0.96~1.22)	1.07 (0.94~1.21)	1.07 (0.94~1.21)

*Ref* Reference

Model 1: Adjusted age, sex, and race

Model 2: Adjusted Model 1+townsend deprivation index, education, BMI, smoking status, frequency of alcohol drinking, and physical activity

Model 3: Adjusted Model 2+hypertension, heart attack, angina, stroke, diabetes, hyperlipidemia, diseases of blood and blood-forming organs, chronic diseases involving the immune mechanism, and malignant neoplasms

A U-shaped relationship was observed between SII, PLR, and the risk of AF (Fig. 2D, E, and G; Table 2). For example, in the case of PLR, the lowest HR for incident AF was found in the (100,120) group, which was set as the reference group, while the highest HR was in the ≥250 group, followed by the <80 group: 1.19 (95% CI: 1.10~1.28) for the ≥250 group and 1.08 (95% CI:1.02~1.14) for the <80 group (Table 2).

A U-shaped relationship was also observed between lymphocyte count and LMR levels, and the incidence risk of AF, but the HRs were not significant for higher levels, with only a slightly increasing trend observed (Fig. 2D and H; Table 2).

### Ventricular arrhythmias

**Fig. 3:**
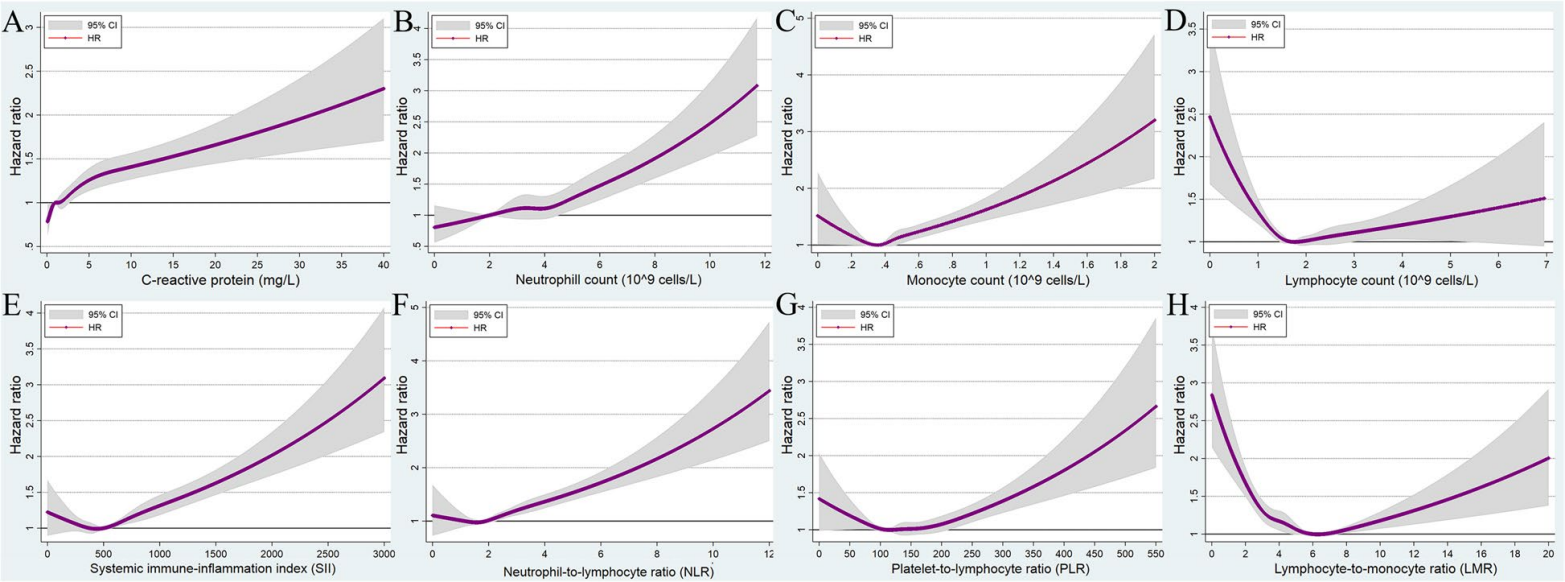
Multivariable-adjusted association between different systematic information indicators and the risk of ventricular arrhythmias by restricted cubic spline regression. **A** C-reactive protein; **B** neutrophil count; **C** monocyte count; **D** lymphocyte count; **E** systemic immune-inflammation index (neutrophils × platelets/lymphocytes); **F** neutrophil-to-lymphocyte ratio (neutrophils/lymphocytes); **G** platelet-to-lymphocyte ratio (platelets/lymphocytes); **H** lymphocyte-to-monocyte ratio (lymphocytes/monocytes). HR, hazard ratio; CI, confidence intervals

There were 3789 VA events across 5.99 million person-years of follow-up (incidence rate: 0.63 events per 1000 person-years, 95% CI: 0.61–0.65). Figure 3 and Table 2 show the association between systemic inflammation indicator levels and VA risk. The relationship between systemic inflammation indicator levels and VA risk was stronger than the relationship between systemic inflammation indicator levels and AF risk.

In general, the risk of incident VA increased monotonically with an increase in the CRP level and neutrophil count (fully adjusted model: HRs = 1.00, 1.06, 1.20, 1.13, 1.28, 1.45, and 1.87 for the increasing CRP groups; HR = 1.00, 1.10, 1.14, 1.17, 1.43, 1.57, and 2.13 for the neutrophil count groups; Figs. 3A and 2B; Table 2). For monocyte count, the HRs for incident VA remained at around 1 in the first five groups (<0.7 × 10^9^ cells/L) and subsequently increased to 1.27 (95% CI: 1.06–1.53) in the (0.7, 0.8) ×10^9^ cells/L group and 1.58 (95% CI: 1.33–1.89) in the ≥0.8 × 10^9^ cells/L group (Table 2). A similar association was observed between NLR and the occurrence of VA.

A U-shaped association was observed between lymphocyte count, SII, PLR, LMR level, and risk of incident VA (Fig. 3D, E, F, and H). For example, compared with individuals with lymphocyte count at the mid-level ((2.0, 2.5) ×10^9^ cells/L) at baseline, individuals with the lowest (<0.8 × 10^9^ cells/L) and highest (≥4.0 × 10^9^ cells/L) lymphocyte levels had a 1.28 (95% CI: 0.92~1.77) and 1.39 (95% CI: 0.99~1.95) times higher chance of being diagnosed with VA during follow-up (Table 2).

### Bradyarrhythmia

**Fig. 4:**
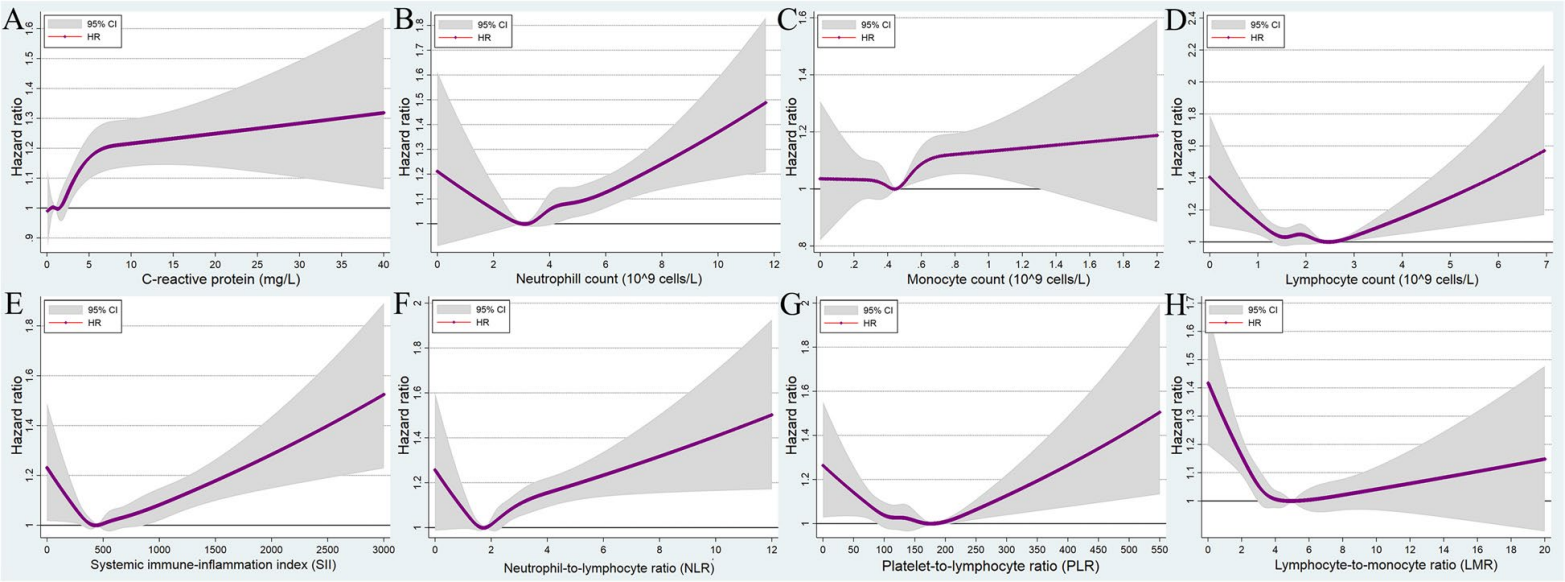
Multivariable-adjusted association between different systematic information indicators and the risk of bradyarrhythmia by restricted cubic spline regression. **A** C-reactive protein; **B** neutrophil count; **C** monocyte count; **D** lymphocyte count; **E** systemic immune-inflammation index (neutrophils × platelets/lymphocytes); **F** neutrophil-to-lymphocyte ratio (neutrophils/lymphocytes); **G** platelet-to-lymphocyte ratio (platelets/lymphocytes); **H** lymphocyte-to-monocyte ratio (lymphocytes/monocytes). HR, hazard ratio; CI, confidence intervals

There were 10,527 incident bradyarrhythmia events across 5.95 million person-years of follow-up (incidence rate: 1.77 events per 1000 person-years, 95% CI: 1.73–1.80). After fully adjusting for covariates, the association of systemic inflammation with the risk of bradyarrhythmia was moderate, and not as strong as its association with AF and VA risk. There was a significant positive correlation between CRP level and incident bradyarrhythmia (Fig. 4A and Table 2). Compared to participants with a lower CRP level (<0.5 mg/L), HR was 1.15 (95% CI: 1.05–1.27) for the (3.0, 4.0) mg/L group, 1.18 (95% CI: 1.08–1.29) for the (4.0, 10.0) mg/L group, and 1.3 (95% CI: 1.16–1.45) for the ≥10.0 mg/L group (Table 2). Overall, the HR for incident bradyarrhythmia tended to increase with an increase in the neutrophil count, monocyte count, and NLR level, after showing a slightly decreasing trend at low neutrophil, monocyte, and NLR levels (Fig. 4B, C, and F; Table 2). Moderate U-shaped correlations were observed between lymphocyte count, SII, PLR, and LMR levels, and HR for incident bradyarrhythmia (Fig. 4D, E, F, and H; Table 2).

### Subgroup analyses

Malignant neoplasms, DBBF, and chronic diseases involving the immune mechanism are three major diseases that may have direct effects on blood cell counts and CRP levels. Heart disease, hypertension, and hyperlipidemia are diseases that may have an impact on the outcome of arrhythmias. Subgroup analysis was therefore performed for the above populations, except for the DBBF group, for which we performed a sensitivity analysis because of its small sample size. In order to assess potential effect modification by age at baseline and sex, we further conducted subgroup analysis by age groups at baseline (less than 60 years old vs. 60 years old or older) and sex.

In all subgroups, the associations between systemic inflammation indicators and the risk of arrhythmia outcomes were similar to those in the overall population analysis described above, although in some cases the trends were not statistically significant (Additional file 1: Tables S4–7). The levels of CRP and several composite inflammatory indicators, such as SII, NLR, PLR, and LMR, were more closely associated with arrhythmia risk than single-cell count markers such as lymphocyte, neutrophil, and monocyte. Further, the associations between systemic inflammation indicators and VA and AF were stronger than the association between the systemic inflammation indicators and bradyarrhythmia.

### Sensitivity analysis

Excluding arrhythmic events that occurred within the first 2 years of follow-up did not obviously alter the results (Additional file 1: Table S8). Excluding participants with malignant neoplasms, DBBF, chronic diseases involving the immune mechanism, and heart diseases, did not alter the results either (Additional file 1: Table S9).

## Discussion

The present study has several noteworthy findings: First, with regard to the relationship between the levels of systemic inflammation indicators and the risk of various arrhythmias, CRP showed a linear positive correlation; monocyte count, neutrophil count, and NLR showed a nonlinear positive correlation; lymphocyte count, SII, PLR, and LMR showed a U-shaped association. Second, after fully adjusting for covariates, the above association still existed and was strongest for VA, followed by AF and bradyarrhythmia. Third, the above trends were further validated in different populations through subgroup analyses and sensitivity analyses.

Although previous studies have shown that elevated CRP is associated with the occurrence and recurrence of AF [12, 21, 22] and an increased risk of malignant VA [23, 24] in populations with structural heart diseases, we validated the exact linear correlation between CRP and various arrhythmias in a notably larger population. Importantly, even after adjusting for all potential confounders, in various subgroup analyses and sensitivity analyses, this linear correlation was sustained and robust and was the strongest among the selected inflammation indicators. A recent study suggested that CRP not only is an inflammatory marker, but also promotes inflammation and subsequent myocardial fibrosis through the TLR4/NF-κB/TGF-β pathway [25]. Another study showed that CRP played a proarrhythmic role by directly affecting calcium homeostasis in cardiomyocytes [26]. Our findings corroborate previous studies and confirm that CRP is a key indicator of arrhythmia risk.

The results of previous studies on the association between differential leukocyte counts and incident AF have been conflicting and not fully consistent with our findings. For example, one study reported a positive association between total leukocyte count and the risk of AF in 6315 individuals, but no association was observed between differential leukocyte counts and the risk of AF [15]. A community-based cohort study with a larger sample of 14,500 participants reported that the total leukocyte, neutrophil, and monocyte counts were positively associated with higher AF risk, while the lymphocyte counts were inversely associated [27]. This inverse association between lymphocyte counts and incident AF differs from the U-shaped association observed in our study, and it might be explained by the elimination of extreme values in their study. The potential associations between differential leukocyte counts and the risk of other types of arrhythmias have been poorly studied. Herein we confirmed the precise non-linear positive correlation between neutrophil and monocyte counts and a U-shaped association for lymphocyte counts in the case of various arrhythmias. This correlation was not only found for AF, but also found for VA and bradyarrhythmia.

The mechanisms by which leukocytes contribute to arrhythmias are complicated. Inflammatory cells infiltrate the myocardium and release reactive oxygen species, cytokines, myeloperoxidase, and hydrolase, leading to irregular interstitial fibrosis that causes electrical and structural remodeling of atrial and ventricular tissue, and consequently, the development of AF and VA [28–31]. Active adhesion and recruitment of inflammatory cells were observed in the atrial tissue of AF patients, and the involved cells included neutrophils, lymphocytes, monocytes, macrophages, and granulocytes [32–34]. The overproduction of inflammatory cytokines by persistent host inflammatory response can also act on the sinoatrial node and cause bradyarrhythmia [35]. However, bradyarrhythmia is mainly caused by sinoatrial/atrioventricular node dysplasia and degeneration. VA/AF are mainly associated with remodeling and sympathetic activation. Inflammation plays an important role in remodeling and sympathetic excitation, and a relatively mild role in dysplasia and degeneration. This may be the mechanistic explanation for the weaker association between systemic inflammation and bradyarrhythmia than VA/AF. The U-shaped association observed between lymphocyte counts and the incidence of arrhythmias also deserves our attention, which may be explained by the physiological stress and inflammatory states under abnormal (both high and low) lymphocyte counts [36].

NLR, PLR, LMR, and SII are composite inflammatory markers derived from ratios of differential leukocyte counts and platelets. They are believed to better reflect the intensity of systemic inflammation and are potentially superior to simple WBC counts [18–20, 37–39]. Our findings confirm this viewpoint. In the subgroup and sensitivity analyses, composite inflammatory markers were more frequently significantly associated with arrhythmia risk than simple blood cell counts. Previous researches on the relationship between these composite markers and AF recurrence/onset have reported conflicting results [13, 14, 38, 40]. Furthermore, the potential relationship between composite markers and other types of arrhythmias remains unresolved and is rarely reported [41]. However, in this large cohort study, we have provided compelling results and demonstrated the exact association between these composite markers and different arrhythmias. Most of the correlation curves were U-shaped and can be explained by the original U-shaped curve that reflects simple lymphocyte.

Considering the character as continuous variables of inflammation indicators, it is not easy to obtain the exact proportion of population at a clinically increase in arrhythmia risk due to inflammation. But this study provides the values of specific risk for arrhythmia onset and will be helpful to provide reference indicators for constructing predictive models for arrhythmia occurrence. Since inflammation plays a prominent role in the development of different arrhythmias, anti-inflammatory drugs are likely to improve cardiovascular outcomes. Colchicine, for example, has a variety of anti-inflammatory effects as a safe and well-tolerated treatment for gout. Several clinical trials have demonstrated the protective effect of colchicine in postoperative atrial fibrillation and in post-ablation atrial fibrillation [42, 43]. Anti-inflammatory therapy targeting the interleukin-1β innate immunity pathway with canakinumab led to a significantly lower rate of recurrent cardiovascular events than placebo [44] and its role in preventing arrhythmias is also worthy of expectation. Combined with our study findings, early intervention on the systemic inflammation may be a promising therapy to reduce the occurrence of arrhythmia.

## Strengths and limitations

Our study has several unique advantages. First, the UKB is a large prospective cohort including diversified inflammatory indicators for over 500,000 individuals with over 12 years of follow-up. This makes the present study the largest analysis that provides the highest level of evidence for the association between systemic inflammation and various arrhythmias to date. Second, our investigation links systemic inflammation to three arrhythmia subtypes, namely, AF, VA, and bradyarrhythmia, the latter two of which have been poorly studied. Third, this study included comprehensive measures of systemic inflammation indicators, including CRP and differential leukocyte count, and composite measures such as SII, LMR, NLR, and PLR.

There are also some limitations to this study. First, this is an observational study and, therefore, cannot prove a causal relationship between systemic inflammation and cardiac arrhythmias. Second, the inflammatory indicators and confounding variables were only assessed at the baseline, and relevant information was lacking during follow-up. That is, these values could have changed over time, but we were unable to document or assess this. Third, we used data from reports of hospitalizations and deaths to diagnose the incidence of arrhythmias, and this may have led to an underestimation of the true incidence, given the likelihood of subclinical onset of arrhythmias. However, to make the study more clinically relevant, we included a diagnosis of arrhythmias that may cause serious adverse outcomes which are not usually followed by subclinical episodes of arrhythmias. Fourth, although we carefully adjusted for various major confounders, biases resulting from unknown and unmeasured confounders may still exist. Finally, this group included individuals of European origin and was mainly a white British population; this limits the applicability of the findings to populations belonging to other races.

## Conclusion

This large-scale prospective study demonstrates that systemic inflammation levels are significantly associated with the risk of cardiac arrhythmias, and the above association is strongest for VA, followed sequentially by AF and bradyarrhythmia. Given the high morbidity and mortality and potential reversibility of severe arrhythmias, early prevention is critical. This study helps to provide reference indicators for constructing predictive models for arrhythmia occurrence in the future. Furthermore, aggressive management of systemic inflammation might have favorable effects on reducing the arrhythmia burden, which need to be further confirmed by randomized controlled studies.

## Supplementary Information

**Additional file 1: Table S1.** Arrhythmia Definitions using the UK biobank. **Table S2.** Definitions of diseases history in the UK Biobank. **Table S3.** Demographic and clinical characteristics of participants in a study of arrhythmias in the UK Biobank. **Table S4.** The hazard risk (HR) with 95% confidence intervals (95%CI) between various systematic information indicators and three arrhythmia subtypes among participants with heart diseases, hypertension, or hyperlipidemia at baseline by the Cox proportional hazard model. **Table S5.** The hazard risk (HR) with 95% confidence intervals (95%CI) between various systematic information indicators and three arrhythmia subtypes among participants with chronic diseases involving the immune mechanism, or malignant neoplasms at baseline by the Cox proportional hazard model. **Table S6.** The hazard risk (HR) with 95% confidence intervals (95%CI) between various systematic information indicators and three arrhythmia subtypes stratified by age group at baseline using the Cox proportional hazard model. **Table S7.** The hazard risk (HR) with 95% confidence intervals (95%CI) between various systematic information indicators and three arrhythmia subtypes stratified by sex using the Cox proportional hazard model. **Table S8.** The hazard risk (HR) with 95% confidence intervals (95%CI) between various systematic information indicators and three arrhythmia subtypes excluding incident cases occurred in the first 2 years of follow-up by the Cox proportional hazard model. **Table S9.** The hazard risk (HR) with 95% confidence intervals (95%CI) between various systematic information indicators and three arrhythmia subtypes excluding participants with heart diseases, diseases of blood and blood-forming organs, chronic diseases involving the immune mechanism, or malignant neoplasms at baseline by the Cox proportional hazard model.

## Data Availability

All data were available in the UK Biobank and subject to registration and application processes.

## Abbreviations

AF: Atrial fibrillation/flutter
BMI: Body mass index
CI: Confidence interval
CRP: C-reactive protein
DBBF: Diseases of blood and blood-forming organs
HR: Hazard ratio
ICD-10: International Statistical Classification of Diseases and Related Health Problems, Tenth Revision
ICD-9: International Classification of Diseases, Ninth Revision
LMR: Lymphocyte-to-monocyte ratio
NLR: Neutrophil-to-lymphocyte ratio
PLR: Platelet-to-lymphocyte ratio
SCD: Sudden cardiac death
SII: Systemic immune-inflammation index
STROBE: Strengthening the Reporting of Observational Studies in Epidemiology
UKB: UK Biobank
VA: Ventricular arrhythmia
