# Supplementary material for: Systemic inflammation indicators and risk of incident arrhythmias in 478,524 individuals: evidence from the UK Biobank cohort
Source: BMC Med. 2023 Feb 28;21:76. doi: 10.1186/s12916-023-02770-5 (PMC9976398; doi:10.1186/s12916-023-02770-5)
Supplement: Supplementary file 1 — Additional file 1: Table S1. Arrhythmia Definitions using the UK biobank. Table S2. Definitions of diseases history in the UK Biobank. Table S3. Demographic and clinical characteristics of participants in a study of arrhythmias in the UK Biobank. Table S4. The hazard risk (HR) with 95% confidence intervals (95%CI) between various systematic information indicators and three arrhythmia subtypes among participants with heart diseases, hypertension, or hyperlipidemia at baseline by the Cox proportional hazard model. Table S5. The hazard risk (HR) with 95% confidence intervals (95%CI) between various systematic information indicators and three arrhythmia subtypes among participants with chronic diseases involving the immune mechanism, or malignant neoplasms at baseline by the Cox proportional hazard model. Table S6. The hazard risk (HR) with 95% confidence intervals (95%CI) between various systematic information indicators and three arrhythmia subtypes stratified by age group at baseline using the Cox proportional hazard model. Table S7. The hazard risk (HR) with 95% confidence intervals (95%CI) between various systematic information indicators and three arrhythmia subtypes stratified by sex using the Cox proportional hazard model. Table S8. The hazard risk (HR) with 95% confidence intervals (95%CI) between various systematic information indicators and three arrhythmia subtypes excluding incident cases occurred in the first 2 years of follow-up by the Cox proportional hazard model. Table S9. The hazard risk (HR) with 95% confidence intervals (95%CI) between various systematic information indicators and three arrhythmia subtypes excluding participants with heart diseases, diseases of blood and blood-forming organs, chronic diseases involving the immune mechanism, or malignant neoplasms at baseline by the Cox proportional hazard model. [file 12916_2023_2770_MOESM1_ESM.docx]

**Systemic inflammation indicators and risk of incident arrhythmias in 478,524 individuals: Evidence from the UK Biobank cohort**

**List of 9 supplementary tables**

**Table S1.** Arrhythmia Definitions using the UK biobank.

**Table S2.** Definitions of diseases history in the UK Biobank.

**Table S3.** Demographic and clinical characteristics of participants in a study of arrhythmias in the UK Biobank.

**Table S4.** The hazard risk (HR) with 95% confidence intervals (95%CI) between various systematic information indicators and three arrhythmia subtypes among participants with heart diseases, hypertension, or hyperlipidemia at baseline by the Cox proportional hazard model.

**Table S5.** The hazard risk (HR) with 95% confidence intervals (95%CI) between various systematic information indicators and three arrhythmia subtypes among participants with chronic diseases involving the immune mechanism, or malignant neoplasms at baseline by the Cox proportional hazard model.

**Table S6.** The hazard risk (HR) with 95% confidence intervals (95%CI) between various systematic information indicators and three arrhythmia subtypes stratified by age group at baseline using the Cox proportional hazard model.

**Table S7.** The hazard risk (HR) with 95% confidence intervals (95%CI) between various systematic information indicators and three arrhythmia subtypes stratified by sex using the Cox proportional hazard model.

**Table S8.** The hazard risk (HR) with 95% confidence intervals (95%CI) between various systematic information indicators and three arrhythmia subtypes excluding incident cases occurred in the first 2 years of follow-up by the Cox proportional hazard model.

**Table S9.** The hazard risk (HR) with 95% confidence intervals (95%CI) between various systematic information indicators and three arrhythmia subtypes excluding participants with heart diseases, diseases of blood and blood-forming organs, chronic diseases involving the immune mechanism, or malignant neoplasms at baseline by the Cox proportional hazard model.

| **Table S1.** Arrhythmia Definitions using the UK biobank | | | |
| --- | --- | --- | --- |
| **Arrhythmia** | **Field Name (Field ID)** | **Code** | **Definition** |
| Atrial Fibrillation/Flutter | Non-cancer illness code, self-reported (20002) | 1471,1483, 1484, 1485, 1486, 1887, | Atrial fibrillation, atrial flutter; wolff parkinson white / wpw syndrome; irregular heart beat; sick sinus syndrome; svt / supraventricular tachycardia |
|  | Diagnoses - ICD10 (41270) | I48, I48.1, I48.2,  I48.3, I48.4, I48.9 | Atrial fibrillation and flutter; paroxysmal atrial fibrillation; persistent atrial fibrillation; chronic atrial fibrillation; typical atrial flutter; atypical atrial flutter; atrial fibrillation and atrial flutter, unspecified. |
|  | Underlying (primary) cause of death: ICD10 (40001) |  |  |
|  | Contributory (secondary) causes of death: ICD10 (40002) |  |  |
|  | Operative procedures - OPCS4 (41272) | K62.1, K62.2, K62.3, K62.4 | Percutaneous transluminal ablation of pulmonary vein to left atrium conducting system; Percutaneous transluminal ablation of atrial wall for atrial flutter; Percutaneous transluminal ablation of conducting system of heart for atrial flutter NEC; Percutaneous transluminal internal cardioversion NEC |
| Ventricular Arrhythmias | Non-cancer illness code, self-reported (20002) | 1077 | Heart arrhythmia |
|  | Diagnoses - ICD10 (41270) | I47.0, I47.2, I49.0, I46.0, I46.1, I46.9 | Re-entry ventricular arrhythmia; Ventricular tachycardia; Ventricular fibrillation and flutter; Cardiac arrest with successful resuscitation; Sudden cardiac death, so described; Cardiac arrest, unspecified |
|  | Underlying (primary) cause of death: ICD10 (40001) |  |  |
|  | Contributory (secondary) causes of death: ICD10 (40002) |  |  |
|  | Operative procedures - OPCS4 (41272) | K57.6, K64.1, X50.3, X50.4, X50.8, X50.9 | Percutaneous transluminal ablation of ventricular wall; Percutaneous radiofrequency ablation of epicardium; Advanced cardiac pulmonary resuscitation; External ventricular defibrillation; Other specified external resuscitation, Unspecified external resuscitation |
| Bradyarrhythmias | Diagnoses - ICD10 (41270) | I44.0, I44.1, I44.2, I44.3, I44.5, I49.5 | Atrioventricular and left bundle-branch block; Atrioventricular block, second degree; Atrioventricular block, complete; Other and unspecified atrioventricular block; Other specified heart block; Sick sinus syndrome |
|  | Underlying (primary) cause of death: ICD10 (40001) |  |  |
|  | Contributory (secondary) causes of death: ICD10 (40002) |  |  |
|  | Operative procedures - OPCS4 (41272) | K60, K60.1, K60.2, K60.3, K60.4, K60.5, K60.6, K60.8, K60.9, K61, K61.1, K61.2, K61.3, K61.4, K61.5, K61.6, K61.8, K61.9 | Cardiac pacemaker system introduced through vein, Implantation of intravenous cardiac pacemaker system NEC, Resiting of lead of intravenous cardiac pacemaker system, Renewal of intravenous cardiac pacemaker system, Removal of intravenous cardiac pacemaker system, Implantation of intravenous single chamber cardiac pacemaker system, Implantation of intravenous dual chamber cardiac pacemaker system, Other specified cardiac pacemaker system introduced through vein, Unspecified cardiac pacemaker system introduced through vein, Other cardiac pacemaker system, Implantation of cardiac pacemaker system NEC, Resitting of lead of cardiac pacemaker system NEC, Renewal of cardiac pacemaker system NEC, Removal of cardiac pacemaker system NEC, Implantation of single chamber cardiac pacemaker system, Implantation of dual chamber cardiac pacemaker system, Other specified other cardiac pacemaker system, Unspecified other cardiac pacemaker system |
| Note: Patients with baseline arrhythmia were excluded by two methods. The first identification method was through baseline self-reported diseases, namely, non-cancer disease code, self-reported (Field: 20002). Another approach was linked to the medical encounter (Field: 41270, 40001, 40002, and 41272). If the diagnosis date of any of the arrhythmia disease types was no later than the enrollment date, these populations were still considered to have a baseline arrhythmia. On the contrary, if the diagnosis date is later than the enrollment date, these populations will be considered as new arrhythmia patients. | | | |

**Table S2.** Definitions of diseases history in the UK Biobank

| **Comorbidity** | **Data field** | **Field name** | **Data codes** |
| --- | --- | --- | --- |
| **Hypertension** | 20002 | Non-cancer illness code, self-reported | 1065, 1072, 1073 |
|  | 6150 | Vascular/heart problems diagnosed by doctor | 4 |
|  | 41270 | Diagnoses - ICD10 | I10*, I11*, I12*, I13*, I15* |
|  | 41271 | Diagnoses – ICD9 | 401-405 |
| **Heart attack/myocardial infarction** | 20002 | Non-cancer illness code, self-reported | 1075 |
|  | 6150 | Vascular/heart problems diagnosed by doctor | 1 |
|  | 41270 | Diagnoses - ICD10 | I21*, I22*, I23* |
|  | 41271 | Diagnoses – ICD9 | 410-412 |
| **Angina** | 20002 | Non-cancer illness code, self-reported | 1074 |
|  | 6150 | Vascular/heart problems diagnosed by doctor | 2 |
|  | 41270 | Diagnoses - ICD10 | I20* |
|  | 41271 | Diagnoses – ICD9 | 413 |
| **Heart diseases** | 20002 | Non-cancer illness code, self-reported | 1074-1080, 1483-1490, 1584-1590, 1426, 1471 |
|  | 6150 | Vascular/heart problems diagnosed by doctor | 1, 2 |
|  | 41270 | Diagnoses - ICD10 | I01*, I05*, I06*, I07*, I09*, I11*, I13*, I2*, I3*, I4*, I5* |
|  | 41271 | Diagnoses – ICD9 | 391, 393-398, 402, 404, 410-414, 415-417, 420-429 |
| **Stroke** | 20002 | Non-cancer illness code, self-reported | 1081, 1086, 1491, 1583 |
|  | 6150 | Vascular/heart problems diagnosed by doctor | 3 |
|  | 41270 | Diagnoses - ICD10 | I64* |
|  | 41271 | Diagnoses – ICD9 | 430-438 |
| **Diabetes** | 20002 | Non-cancer illness code, self-reported | 1220-1223, 1521 |
|  | 41270 | Diagnoses - ICD10 | E10*, E11*, E12*, E13*, E14* |
|  | 41271 | Diagnoses – ICD9 | 250 |
| **Hyperlipidemia** | 20002 | Non-cancer illness code, self-reported | 1473 |
|  | 41270 | Diagnoses - ICD10 | E78.0, E78.1, E78.2, E78.4, E78.5 |
|  | 41271 | Diagnoses – ICD9 | 272 |
| **Diseases of blood and blood-forming organs (DBBF)** | 20002 | Non-cancer illness code, self-reported | 1327, 1331, 1332, 1447-1449, 1546 |
|  | 41270 | Diagnoses - ICD10 | D61*, D69*, D7* |
|  | 41271 | Diagnoses – ICD9 | 280-289 |
| **Chronic diseases involving the immune mechanism** | 20002 | Non-cancer illness code, self-reported | 1222, 1225, 1260, 1261, 1313, 1322, 1371-1374, 1376-1387, 1428, 1437, 1453, 1454, 1456, 1461-1464, 1475, 1477, 1480, 1481, 1506, 1522, 1564, 1661 |
|  | 41270 | Diagnoses - ICD10 | D8*, M3*, D510*, E06*, E07*, E10*, G35*, G700*, K900*, K50*, K51*, L40*, M45*, M07*, M05*, M06*, L93*, L80*, |
|  | 41271 | Diagnoses – ICD9 | 242, 245, 250, 340, 358, 555, 556, 696, 709, 710, 714, 720 |
| **Malignant neoplasms** | 20001 | Cancer code, self-reported | ≥1 |
|  | 41270 | Diagnoses - ICD10 | C* |
|  | 41271 | Diagnoses – ICD9 | 140-209 |

*wildcard character

Patients with baseline diseases were identified by two methods. The first identification method was through baseline self-reported diseases, namely, self-reported disease codes (Field: 20002, 20001 and 6150). Another approach was linked to the medical encounter (Field: 41270 and41271). If the diagnosis date of any of the specific disease type was no later than the enrollment date, these populations were still considered to have the corresponding baseline diseases.

| **Table S3. Demographic and clinical characteristics of participants in a study of arrhythmias in the UK Biobank (N=457426)** | | | | |
| --- | --- | --- | --- | --- |
| **Characteristic** | **CRP-low**  **(< 1.0 mg/L) N=180,147** | **CRP-middle**  **(1.0 to 3.0 mg/L) N=174,093** | **CRP-high**  **(> 3.0 mg/L) N=103,186** | **P value** |
| **Age (years)** | 55.29±8.16 | 57.07±7.98 | 57.32±7.91 | <0.0001 |
| **Female, n (%)** | 96906(53.79) | 91625(52.63) | 61520(59.62) | <0.0001 |
| **White race, n (%)** | 169915(94.73) | 164308(94.84) | 96680(94.18) | <0.0001 |
| **Townsend deprivation index** | -1.53±2.97 | -1.34±3.07 | -0.88±3.26 | <0.0001 |
| **Educational level, n (%)** |  |  |  |  |
| College degree | 70350(39.44) | 52531(30.54) | 25640(25.23) | <0.0001 |
| High school graduate | 21545(12.08) | 18891(10.98) | 10589(10.42) |  |
| Middle school graduate | 36562(20.5) | 37903(22.04) | 22436(22.08) |  |
| None of the above | 49934(27.99) | 62658(36.43) | 42967(42.28) |  |
| **Body mass index (kg/m2)** | 25.27±3.55 | 27.87±4.2 | 30.29±5.69 | <0.0001 |
| **Smoking status, n (%)** |  |  |  |  |
| Never | 106233(59.21) | 93417(53.93) | 50336(49.11) | <0.0001 |
| Former | 58514(32.61) | 61332(35.41) | 37016(36.11) |  |
| Current | 14666(8.17) | 18455(10.66) | 15153(14.78) |  |
| **Daily alcohol, n (%)** |  |  |  |  |
| Daily or almost daily | 39672(22.06) | 35263(20.3) | 17816(17.32) | <0.0001 |
| Three or four times a week | 46361(25.78) | 39425(22.69) | 19875(19.32) |  |
| Once or twice a week | 46463(25.84) | 45570(26.23) | 26153(25.42) |  |
| Less once a week | 35395(19.68) | 39770(22.89) | 28266(27.48) |  |
| Never | 11940(6.64) | 13694(7.88) | 10758(10.46) |  |
| **Physical activity, n (%)** |  |  |  |  |
| Light | 56921(32.84) | 58747(35.67) | 37937(39.81) | <0.0001 |
| Moderate | 44444(25.64) | 41631(25.28) | 22473(23.58) |  |
| High | 71975(41.52) | 64315(39.05) | 34883(36.61) |  |
| **Disease history at baseline** |  |  |  |  |
| Hypertension | 37787(20.98) | 51636(29.66) | 38282(37.1) | <0.0001 |
| Heart attack | 3377(1.87) | 3729(2.14) | 2485(2.41) | <0.0001 |
| Angina | 5495(3.05) | 6615(3.8) | 4777(4.63) | <0.0001 |
| Stroke | 2215(1.23) | 2709(1.56) | 2119(2.05) | <0.0001 |
| Diabetes | 6968(3.87) | 9381(5.39) | 7775(7.54) | <0.0001 |
| Hyperlipidemia | 21617(12) | 25091(14.42) | 15028(14.57) | <0.0001 |
| Diseases of blood and blood-forming organs (DBBF) | 1318(0.73) | 1527(0.88) | 1275(1.24) | <0.0001 |
| Chronic diseases involving the immune mechanism | 21891(12.15) | 23237(13.35) | 17480(16.95) | <0.0001 |
| Malignant neoplasms | 13886(7.71) | 15458(8.88) | 10461(10.14) | <0.0001 |
| **Systemic inflammation indicators** |  |  |  |  |
| C-reactive protein (CRP, mg/L) | 0.56±0.24 | 1.77±0.56 | 7.51±7.09 | <0.0001 |
| Neutrophil count (10^9 cells/L) | 3.86±1.23 | 4.23±1.33 | 4.83±1.62 | <0.0001 |
| Monocyte count (10^9 cells/L) | 0.44±0.33 | 0.48±0.21 | 0.52±0.25 | <0.0001 |
| Lymphocyte count (10^9 cells/L) | 1.89±1.36 | 1.99±0.98 | 2.06±1.03 | <0.0001 |
| Systemic immune-inflammation index (SII) | 547.6±305.96 | 590.47±345.65 | 701.56±451.93 | <0.0001 |
| Neutrophil-to-lymphocyte ratio (NLR) | 2.23±1.08 | 2.32±1.21 | 2.61±1.46 | <0.0001 |
| Platelet-to-lymphocyte ratio (PLR) | 142.51±59.02 | 140.05±79.65 | 145.13±67.61 | <0.0001 |
| Lymphocyte-to-monocyte ratio (LMR) | 4.69±4 | 4.62±4.77 | 4.56±3.76 | <0.0001 |
| **Follow-up years (years)** | 12.4±2.06 | 12.21±2.36 | 11.94±2.74 | <0.0001 |

**Table S4.** The hazard risk (HR) with 95% confidence intervals (95%CI) between various systematic information indicators and three arrhythmia subtypes among participants with heart diseases, hypertension, or hyperlipidemia at baseline by the Cox proportional hazard model.

| Variables | Participants with heart diseases at baseline ^a^ | | | | | | | | Participants with hypertension at baseline ^b^ | | | Participants with hyperlipidemia at baseline ^c^ | | |
| --- | --- | --- | --- | --- | --- | --- | --- | --- | --- | --- | --- | --- | --- | --- |
|  | **Atrial Fibrillation**  **/Flutter** | **Ventricular Arrhythmia** | | | **Bradyarrhythmia** | | | **Atrial Fibrillation**  **/Flutter** | | **Ventricular Arrhythmia** | **Bradyarrhythmia** | **Atrial Fibrillation**  **/Flutter** | **Ventricular Arrhythmia** | **Bradyarrhythmia** |
| C-reactive protein (mg/L) | | | |  | | | | |  |  |  |  |  |  |
| <0.5 | 1.00 (Ref.) | 1.00 (Ref.) | | | 1.00 (Ref.) | | | 1.00 (Ref.) | | 1.00 (Ref.) | 1.00 (Ref.) | 1.00 (Ref.) | 1.00 (Ref.) | 1.00 (Ref.) |
| [0.5, 1.0) | 0.99(0.88~1.11) | 0.86(0.66~1.1) | | | 1.05(0.9~1.22) | | | 0.97(0.9~1.05) | | 0.96(0.78~1.18) | 0.99(0.88~1.12) | 0.99(0.89~1.1) | 0.8(0.62~1.02) | 1.01(0.88~1.16) |
| [1.0, 2.0) | 1.05(0.95~1.17) | 1.05(0.82~1.33) | | | 1.12(0.96~1.3) | | | 1.03(0.96~1.12) | | 1.1(0.91~1.34) | 0.99(0.88~1.11) | 1.01(0.92~1.12) | 0.99(0.79~1.25) | 0.95(0.83~1.1) |
| [2.0, 3.0) | 1.1(0.98~1.24) | 0.98(0.74~1.29) | | | 1.07(0.9~1.27) | | | 1.07(0.98~1.16) | | 0.96(0.77~1.2) | 1.09(0.97~1.24) | 1.07(0.95~1.19) | 0.74(0.56~0.99) | 1.11(0.95~1.29) |
| [3.0, 4.0) | 1.03(0.9~1.19) | 1.15(0.84~1.58) | | | 1.17(0.96~1.43) | | | 1.06(0.97~1.17) | | 1.28(1.01~1.62) | 1.15(1~1.32) | 1.13(0.99~1.29) | 1.07(0.79~1.44) | 1.17(0.98~1.4) |
| [4.0, 10.0) | 1.14(1.01~1.29) | 1.29(0.99~1.7) | | | 1.23(1.03~1.46) | | | 1.17(1.08~1.28) | | 1.43(1.16~1.77) | 1.19(1.05~1.35) | 1.19(1.06~1.33) | 1.19(0.91~1.55) | 1.13(0.96~1.33) |
| >=10.0 | 1.4(1.21~1.62) | 1.54(1.11~2.14) | | | 1.29(1.03~1.61) | | | 1.35(1.22~1.49) | | 1.71(1.33~2.19) | 1.31(1.13~1.53) | 1.31(1.13~1.51) | 1.35(0.96~1.88) | 1.25(1.01~1.54) |
| Neutrophil count (10^9 cells/L) | | | | | |  | | |  |  |  |  |  |  |
| <2.0 | 1.00 (Ref.) | 1.00 (Ref.) | | | 1.00 (Ref.) | | | 1.00 (Ref.) | | 1.00 (Ref.) | 1.00 (Ref.) | 1.00 (Ref.) | 1.00 (Ref.) | 1.00 (Ref.) |
| [2.0, 3.0) | 0.95(0.71~1.29) | 0.92(0.46~1.84) | | | 0.85(0.57~1.29) | | | 1.13(0.92~1.38) | | 1.03(0.62~1.72) | 0.92(0.69~1.23) | 1.05(0.78~1.41) | 1.34(0.62~2.91) | 1.03(0.67~1.59) |
| [3.0, 4.0) | 1.02(0.76~1.36) | 0.98(0.5~1.91) | | | 0.96(0.65~1.43) | | | 1.06(0.87~1.29) | | 1.03(0.63~1.68) | 0.99(0.75~1.31) | 1.02(0.76~1.35) | 1.15(0.54~2.45) | 1.12(0.74~1.7) |
| [4.0, 5.0) | 1.01(0.75~1.34) | 1.13(0.58~2.19) | | | 0.97(0.66~1.44) | | | 1.12(0.92~1.37) | | 1.1(0.67~1.79) | 1.06(0.8~1.41) | 1.04(0.78~1.38) | 1.32(0.62~2.81) | 1.16(0.76~1.75) |
| [5.0, 6.0) | 1.1(0.82~1.47) | 1.11(0.57~2.19) | | | 1.05(0.7~1.56) | | | 1.19(0.97~1.45) | | 1.26(0.77~2.06) | 1.09(0.82~1.45) | 1.1(0.82~1.46) | 1.39(0.65~2.97) | 1.21(0.8~1.85) |
| [6.0, 7.5) | 1.17(0.87~1.58) | 1.33(0.67~2.63) | | | 1.15(0.77~1.73) | | | 1.3(1.07~1.6) | | 1.36(0.83~2.25) | 1.22(0.91~1.63) | 1.22(0.91~1.63) | 1.61(0.75~3.46) | 1.35(0.88~2.07) |
| >=7.5 | 1.29(0.94~1.76) | 1.85(0.91~3.73) | | | 1.16(0.75~1.79) | | | 1.48(1.2~1.84) | | 1.96(1.17~3.29) | 1.31(0.96~1.79) | 1.36(1~1.84) | 2.15(0.98~4.72) | 1.45(0.93~2.27) |
| Monocyte count (10^9 cells/L) | | | |  | | | | |  |  |  |  |  |  |
| <0.3 | 1.00 (Ref.) | 1.00 (Ref.) | | | 1.00 (Ref.) | | | 1.00 (Ref.) | | 1.00 (Ref.) | 1.00 (Ref.) | 1.00 (Ref.) | 1.00 (Ref.) | 1.00 (Ref.) |
| [0.3, 0.4) | 0.9(0.77~1.05) | 0.77(0.54~1.09) | | | 0.87(0.7~1.07) | | | 1.01(0.92~1.12) | | 0.93(0.72~1.21) | 0.96(0.83~1.12) | 0.96(0.84~1.11) | 0.69(0.49~0.97) | 1.03(0.84~1.26) |
| [0.4, 0.5) | 0.83(0.72~0.96) | 0.77(0.56~1.07) | | | 0.76(0.62~0.93) | | | 1(0.91~1.1) | | 1.03(0.8~1.31) | 1.02(0.88~1.17) | 0.98(0.86~1.12) | 0.82(0.6~1.12) | 0.99(0.82~1.19) |
| [0.5, 0.6) | 0.91(0.78~1.05) | 0.74(0.53~1.02) | | | 0.83(0.68~1.01) | | | 1.04(0.95~1.15) | | 1.02(0.79~1.3) | 1.06(0.92~1.22) | 0.98(0.86~1.12) | 0.91(0.66~1.24) | 0.98(0.81~1.19) |
| [0.6, 0.7) | 0.96(0.83~1.12) | 0.79(0.57~1.11) | | | 0.79(0.64~0.98) | | | 1.11(1~1.22) | | 1.09(0.84~1.4) | 1.04(0.9~1.21) | 1.08(0.94~1.24) | 0.87(0.63~1.2) | 1(0.82~1.22) |
| [0.7, 0.8) | 0.96(0.82~1.12) | 0.87(0.61~1.25) | | | 0.91(0.73~1.14) | | | 1.15(1.04~1.28) | | 1.16(0.88~1.52) | 1.14(0.97~1.34) | 1.12(0.97~1.3) | 0.83(0.58~1.19) | 1.02(0.82~1.26) |
| >=0.8 | 1.02(0.87~1.2) | 1.16(0.82~1.63) | | | 0.88(0.71~1.1) | | | 1.16(1.05~1.29) | | 1.52(1.17~1.98) | 1.08(0.92~1.27) | 1.11(0.96~1.29) | 1.18(0.85~1.65) | 0.9(0.73~1.12) |
| Lymphocyte count (10^9 cells/L) | | |  | | | | | |  |  |  |  |  |  |
| <0.8 | 1.53(1.17~1.99) | 1.16(0.59~2.26) | | | 1.08(0.69~1.67) | | | 1.49(1.25~1.79) | | 1.38(0.86~2.19) | 1.26(0.95~1.68) | 1.57(1.21~2.04) | 1.19(0.59~2.41) | 1.51(1.04~2.2) |
| [0.8, 1.5) | 1.16(1.07~1.27) | 1.12(0.92~1.37) | | | 1.01(0.89~1.14) | | | 1.15(1.09~1.22) | | 1.22(1.06~1.41) | 0.98(0.9~1.07) | 1.15(1.06~1.24) | 1.2(0.99~1.45) | 1.03(0.92~1.16) |
| [1.5, 2.0) | 1.03(0.96~1.12) | 0.96(0.8~1.14) | | | 1.02(0.91~1.14) | | | 1.05(1~1.1) | | 0.95(0.83~1.09) | 0.99(0.92~1.07) | 1.07(0.99~1.14) | 0.89(0.75~1.07) | 1.02(0.92~1.13) |
| [2.0, 2.5) | 1.00 (Ref.) | 1.00 (Ref.) | | | 1.00 (Ref.) | | | 1.00 (Ref.) | | 1.00 (Ref.) | 1.00 (Ref.) | 1.00 (Ref.) | 1.00 (Ref.) | 1.00 (Ref.) |
| [2.5, 3.0) | 0.9(0.81~1.01) | 0.88(0.69~1.12) | | | 0.94(0.81~1.1) | | | 0.95(0.89~1.02) | | 0.84(0.7~1.01) | 0.95(0.86~1.06) | 0.92(0.84~1.02) | 0.94(0.75~1.19) | 0.91(0.79~1.05) |
| [3.0, 4.0) | 1.02(0.89~1.16) | 0.96(0.71~1.29) | | | 1.16(0.96~1.39) | | | 0.97(0.88~1.05) | | 1.03(0.83~1.28) | 1.12(0.99~1.27) | 0.91(0.8~1.03) | 1.13(0.86~1.48) | 1.06(0.89~1.25) |
| >=4.0 | 1.23(0.92~1.62) | 0.96(0.49~1.88) | | | 1.64(1.15~2.35) | | | 1.05(0.87~1.28) | | 0.75(0.43~1.31) | 1.02(0.75~1.37) | 1.1(0.86~1.42) | 0.96(0.51~1.81) | 0.99(0.68~1.46) |
| Systemic immune-inflammation index (SII) | | | | | | |  | |  |  |  |  |  |  |
| <300 | 1.02(0.91~1.15) | 0.86(0.65~1.15) | | | 1.07(0.91~1.26) | | | 1.05(0.97~1.13) | | 0.97(0.78~1.21) | 1.05(0.93~1.18) | 1.1(0.98~1.22) | 1.08(0.82~1.41) | 1.02(0.87~1.18) |
| [300, 400) | 1.06(0.96~1.18) | 1.12(0.88~1.41) | | | 1(0.87~1.16) | | | 1.05(0.98~1.13) | | 1.09(0.91~1.31) | 1.01(0.91~1.12) | 1.08(0.98~1.18) | 1.17(0.92~1.48) | 0.91(0.79~1.04) |
| [400, 500) | 1.00 (Ref.) | 1.00 (Ref.) | | | 1.00 (Ref.) | | | 1.00 (Ref.) | | 1.00 (Ref.) | 1.00 (Ref.) | 1.00 (Ref.) | 1.00 (Ref.) | 1.00 (Ref.) |
| [500, 600) | 1(0.9~1.1) | 1.12(0.88~1.41) | | | 1.02(0.88~1.18) | | | 1.02(0.95~1.09) | | 1.09(0.91~1.31) | 1(0.9~1.1) | 1.01(0.92~1.12) | 1.2(0.94~1.52) | 0.94(0.82~1.07) |
| [600, 800) | 1.01(0.92~1.11) | 1.12(0.9~1.4) | | | 1.02(0.89~1.17) | | | 1.07(1.01~1.14) | | 1.17(0.99~1.38) | 1.04(0.95~1.15) | 1.14(1.05~1.25) | 1.21(0.97~1.51) | 1.08(0.96~1.22) |
| [800, 1500) | 1.08(0.98~1.19) | 1.04(0.82~1.31) | | | 1.05(0.91~1.21) | | | 1.16(1.09~1.24) | | 1.39(1.17~1.64) | 1.11(1.01~1.22) | 1.22(1.11~1.34) | 1.28(1.02~1.62) | 1.03(0.91~1.18) |
| >=1500 | 1.37(1.15~1.64) | 1.93(1.35~2.78) | | | 1.21(0.92~1.58) | | | 1.52(1.35~1.7) | | 2.46(1.9~3.19) | 1.44(1.2~1.72) | 1.49(1.25~1.78) | 2.12(1.44~3.12) | 1.63(1.28~2.07) |
| Neutrophil-to-lymphocyte ratio (NLR) | | | | | | |  | |  |  |  |  |  |  |
| <1.5 | 1.00 (Ref.) | 1.00 (Ref.) | | | 1.00 (Ref.) | | | 1.00 (Ref.) | | 1.00 (Ref.) | 1.00 (Ref.) | 1.00 (Ref.) | 1.00 (Ref.) | 1.00 (Ref.) |
| [1.5, 2.0) | 1.03(0.93~1.15) | 1.16(0.89~1.51) | | | 0.94(0.81~1.09) | | | 1(0.94~1.08) | | 1.14(0.94~1.38) | 0.96(0.87~1.07) | 0.97(0.88~1.07) | 0.94(0.74~1.2) | 1.02(0.89~1.18) |
| [2.0, 2.5) | 1.08(0.97~1.2) | 1.38(1.07~1.79) | | | 0.97(0.83~1.12) | | | 1.06(0.99~1.13) | | 1.28(1.06~1.55) | 1.01(0.91~1.12) | 1.06(0.96~1.16) | 1.18(0.94~1.5) | 1.12(0.98~1.29) |
| [2.5, 3.0) | 1.19(1.07~1.33) | 1.31(0.99~1.72) | | | 1.04(0.89~1.21) | | | 1.14(1.06~1.23) | | 1.24(1.01~1.51) | 1.05(0.94~1.17) | 1.11(1~1.23) | 1.1(0.85~1.42) | 1.11(0.96~1.29) |
| [3.0, 3.5) | 1.15(1.01~1.3) | 1.56(1.17~2.09) | | | 1.14(0.96~1.34) | | | 1.16(1.07~1.26) | | 1.31(1.05~1.64) | 1.13(1~1.28) | 1.18(1.05~1.32) | 1.08(0.81~1.44) | 1.28(1.09~1.51) |
| [3.5, 5.0) | 1.34(1.19~1.51) | 1.59(1.2~2.12) | | | 0.99(0.83~1.17) | | | 1.36(1.26~1.47) | | 1.71(1.4~2.1) | 1.13(1~1.27) | 1.3(1.17~1.45) | 1.31(1~1.72) | 1.08(0.92~1.28) |
| >=5.0 | 1.42(1.21~1.66) | 1.7(1.18~2.47) | | | 1.12(0.89~1.42) | | | 1.54(1.38~1.71) | | 2.43(1.89~3.11) | 1.29(1.1~1.51) | 1.53(1.32~1.77) | 1.91(1.36~2.67) | 1.46(1.18~1.81) |
| Platelet-to-lymphocyte ratio (PLR) | | | | | | |  | |  |  |  |  |  |  |
| <80 | 1.03(0.92~1.15) | 0.97(0.75~1.24) | | | 0.93(0.8~1.09) | | | 1.1(1.02~1.18) | | 0.98(0.8~1.2) | 1.06(0.95~1.18) | 1.07(0.97~1.19) | 1.08(0.84~1.38) | 0.97(0.84~1.12) |
| [80, 100) | 1.07(0.97~1.19) | 1.11(0.89~1.38) | | | 1.01(0.88~1.16) | | | 1.05(0.99~1.13) | | 1.01(0.85~1.21) | 0.97(0.88~1.07) | 1.04(0.95~1.14) | 1.12(0.9~1.4) | 0.91(0.8~1.03) |
| [100, 120) | 1.00 (Ref.) | 1.00 (Ref.) | | | 1.00 (Ref.) | | | 1.00 (Ref.) | | 1.00 (Ref.) | 1.00 (Ref.) | 1.00 (Ref.) | 1.00 (Ref.) | 1.00 (Ref.) |
| [120, 150) | 0.97(0.88~1.06) | 0.99(0.81~1.22) | | | 0.89(0.79~1.01) | | | 1.04(0.98~1.11) | | 1.09(0.93~1.28) | 0.94(0.86~1.03) | 1.09(1~1.18) | 1.08(0.88~1.33) | 0.92(0.82~1.03) |
| [150, 200) | 0.94(0.85~1.03) | 0.82(0.65~1.03) | | | 0.83(0.73~0.95) | | | 1.04(0.98~1.11) | | 1.05(0.89~1.23) | 0.94(0.86~1.04) | 1.04(0.95~1.14) | 1.04(0.84~1.3) | 0.94(0.83~1.06) |
| [200, 250) | 0.96(0.84~1.1) | 0.93(0.67~1.28) | | | 0.86(0.71~1.05) | | | 1.12(1.03~1.22) | | 1.26(1.01~1.57) | 0.96(0.84~1.09) | 1.22(1.08~1.38) | 1.21(0.89~1.65) | 0.96(0.81~1.16) |
| >=250 | 1.27(1.09~1.48) | 1.56(1.13~2.16) | | | 0.97(0.77~1.23) | | | 1.24(1.12~1.38) | | 1.76(1.39~2.24) | 1.12(0.95~1.31) | 1.11(0.94~1.31) | 1.44(0.99~2.09) | 1.24(0.99~1.54) |
| Lymphocyte-to-monocyte ratio (LMR) | | | | | | |  | |  |  |  |  |  |  |
| <2.5 | 1.43(1.25~1.64) | 1.69(1.2~2.37) | | | 1.04(0.87~1.26) | | | 1.46(1.34~1.6) | | 2(1.57~2.55) | 1.13(0.99~1.29) | 1.41(1.25~1.6) | 1.94(1.37~2.75) | 1.04(0.88~1.24) |
| [2.5, 3.0) | 1.1(0.96~1.27) | 1.87(1.33~2.61) | | | 0.84(0.69~1.02) | | | 1.22(1.11~1.33) | | 1.82(1.42~2.34) | 1.01(0.88~1.15) | 1.18(1.04~1.33) | 2.12(1.5~2.99) | 0.94(0.78~1.12) |
| [3.0, 4.0) | 1.09(0.96~1.24) | 1.45(1.05~2) | | | 0.94(0.79~1.11) | | | 1.16(1.08~1.26) | | 1.47(1.17~1.86) | 1.02(0.91~1.15) | 1.11(0.99~1.24) | 1.61(1.16~2.23) | 0.95(0.81~1.11) |
| [4.0, 5.0) | 1.09(0.95~1.23) | 1.3(0.93~1.8) | | | 0.99(0.83~1.18) | | | 1.06(0.98~1.15) | | 1.39(1.1~1.77) | 1(0.88~1.13) | 0.99(0.88~1.12) | 1.71(1.23~2.38) | 0.96(0.81~1.13) |
| [5.0, 6.0) | 0.93(0.81~1.08) | 1.05(0.73~1.52) | | | 0.8(0.66~0.98) | | | 1.01(0.93~1.11) | | 1.13(0.87~1.47) | 0.89(0.78~1.03) | 1.06(0.93~1.2) | 1.28(0.89~1.85) | 0.93(0.77~1.11) |
| [6.0, 8.0) | 1.00 (Ref.) | 1.00 (Ref.) | | | 1.00 (Ref.) | | | 1.00 (Ref.) | | 1.00 (Ref.) | 1.00 (Ref.) | 1.00 (Ref.) | 1.00 (Ref.) | 1.00 (Ref.) |
| >=8.0 | 1.16(0.96~1.4) | 1.52(0.96~2.4) | | | 1.04(0.79~1.36) | | | 1.1(0.97~1.25) | | 1.13(0.79~1.62) | 1.06(0.88~1.28) | 1.05(0.88~1.26) | 1.97(1.28~3.04) | 1.07(0.84~1.37) |
| Ref., Reference.  a Adjusted age, sex, race, townsend deprivation index, education, BMI, smoking status, frequency of alcohol drinking, physical activity, hypertension, stroke, diabetes, hyperlipidemia, diseases of blood and blood-forming organs, chronic diseases involving the immune mechanism, and malignant neoplasms.  b Adjusted age, sex, race, townsend deprivation index, education, BMI, smoking status, frequency of alcohol drinking, and physical activity, heart attack, angina, stroke, diabetes, hyperlipidemia, diseases of blood and blood-forming organs, chronic diseases involving the immune mechanism, and malignant neoplasms.  c Adjusted age, sex, race, townsend deprivation index, education, BMI, smoking status, frequency of alcohol drinking, physical activity, hypertension, heart attack, angina, stroke, diabetes, diseases of blood and blood-forming organs, chronic diseases involving the immune mechanism, and malignant neoplasms. | | | | | | | | | | | | | | |

**Table S5.** The hazard risk (HR) with 95% confidence intervals (95%CI) between various systematic information indicators and three arrhythmia subtypes among participants with chronic diseases involving the immune mechanism, or malignant neoplasms at baseline by the Cox proportional hazard model.

| Variables | Participants with chronic diseases involving the immune mechanism at baseline ^a^ | | | Participants with malignant neoplasms at baseline ^b^ | | | | | | |
| --- | --- | --- | --- | --- | --- | --- | --- | --- | --- | --- |
|  | **Atrial Fibrillation**  **/Flutter** | **Ventricular Arrhythmia** | **Bradyarrhythmia** | **Atrial Fibrillation**  **/Flutter** | | **Ventricular Arrhythmia** | | **Bradyarrhythmia** | | |
| C-reactive protein (mg/L) | | |  |  | |  | |  | | |
| <0.5 | 1.00 (Ref.) | 1.00 (Ref.) | 1.00 (Ref.) | 1.00 (Ref.) | | 1.00 (Ref.) | | 1.00 (Ref.) | | |
| [0.5, 1.0) | 1.03(0.89~1.18) | 1.24(0.88~1.75) | 1.06(0.85~1.31) | 1(0.86~1.16) | | 1.2(0.77~1.86) | | 0.92(0.73~1.16) | | |
| [1.0, 2.0) | 1.08(0.94~1.23) | 1.09(0.78~1.54) | 1.14(0.92~1.41) | 1.02(0.88~1.18) | | 1.2(0.79~1.84) | | 0.94(0.75~1.18) | | |
| [2.0, 3.0) | 1.15(0.99~1.33) | 1.17(0.8~1.71) | 1.24(0.98~1.56) | 0.99(0.85~1.17) | | 1.52(0.96~2.41) | | 0.96(0.74~1.24) | | |
| [3.0, 4.0) | 1.22(1.04~1.44) | 1.11(0.72~1.72) | 1.3(1.01~1.68) | 1.01(0.84~1.21) | | 1.36(0.8~2.32) | | 1.19(0.89~1.58) | | |
| [4.0, 10.0) | 1.28(1.1~1.48) | 1.58(1.11~2.27) | 1.34(1.07~1.69) | 1.15(0.98~1.35) | | 1.86(1.18~2.94) | | 1.16(0.9~1.51) | | |
| >=10.0 | 1.38(1.16~1.64) | 1.8(1.19~2.71) | 1.37(1.04~1.79) | 1.45(1.2~1.76) | | 2.66(1.59~4.43) | | 1.34(0.98~1.84) | | |
| Neutrophil count (10^9 cells/L) | | |  |  |  | | | |  | |
| <2.0 | 1.00 (Ref.) | 1.00 (Ref.) | 1.00 (Ref.) | 1.00 (Ref.) | | 1.00 (Ref.) | | 1.00 (Ref.) | | |
| [2.0, 3.0) | 0.79(0.59~1.05) | 1.44(0.57~3.6) | 0.67(0.44~1.02) | 1.06(0.75~1.49) | | 1.43(0.51~3.98) | | 0.96(0.57~1.62) | | |
| [3.0, 4.0) | 0.74(0.56~0.96) | 1.4(0.57~3.44) | 0.64(0.43~0.96) | 0.98(0.71~1.37) | | 1.19(0.44~3.26) | | 0.94(0.57~1.55) | | |
| [4.0, 5.0) | 0.84(0.64~1.1) | 1.59(0.65~3.9) | 0.73(0.49~1.09) | 1.04(0.75~1.45) | | 1.49(0.55~4.08) | | 0.87(0.53~1.45) | | |
| [5.0, 6.0) | 0.84(0.64~1.11) | 1.77(0.72~4.38) | 0.79(0.52~1.18) | 1.15(0.82~1.61) | | 1.96(0.71~5.4) | | 0.89(0.53~1.5) | | |
| [6.0, 7.5) | 1.02(0.77~1.35) | 2.12(0.85~5.28) | 0.86(0.56~1.3) | 1.11(0.78~1.58) | | 2.04(0.72~5.72) | | 1.04(0.61~1.77) | | |
| >=7.5 | 1.2(0.89~1.62) | 3.54(1.41~8.93) | 1.04(0.67~1.63) | 1.48(1.02~2.15) | | 1.97(0.65~5.97) | | 0.99(0.54~1.81) | | |
| Monocyte count (10^9 cells/L) | | |  |  | |  | |  | | |
| <0.3 | 1.00 (Ref.) | 1.00 (Ref.) | 1.00 (Ref.) | 1.00 (Ref.) | | 1.00 (Ref.) | | 1.00 (Ref.) | | |
| [0.3, 0.4) | 0.96(0.82~1.12) | 0.72(0.49~1.06) | 0.78(0.61~1) | 0.9(0.76~1.05) | | 1.01(0.6~1.68) | | 0.91(0.69~1.18) | | |
| [0.4, 0.5) | 0.93(0.8~1.08) | 0.92(0.64~1.31) | 0.84(0.67~1.06) | 0.88(0.76~1.03) | | 1.39(0.86~2.23) | | 0.97(0.75~1.25) | | |
| [0.5, 0.6) | 1.02(0.88~1.19) | 0.96(0.67~1.37) | 0.94(0.74~1.18) | 0.91(0.77~1.06) | | 1.17(0.71~1.92) | | 0.79(0.61~1.03) | | |
| [0.6, 0.7) | 1.15(0.98~1.35) | 0.92(0.62~1.36) | 1.06(0.83~1.35) | 1.01(0.85~1.19) | | 1.43(0.85~2.39) | | 1.03(0.78~1.36) | | |
| [0.7, 0.8) | 1.07(0.89~1.28) | 0.86(0.55~1.35) | 1.05(0.8~1.38) | 1.07(0.88~1.3) | | 2.45(1.45~4.13) | | 0.95(0.69~1.31) | | |
| >=0.8 | 1.2(1~1.44) | 1.41(0.94~2.14) | 0.99(0.75~1.31) | 1.15(0.95~1.39) | | 1.76(1.01~3.07) | | 0.99(0.71~1.36) | | |
| Lymphocyte count (10^9 cells/L) | | |  |  | |  | |  | | |
| <0.8 | 1.49(1.17~1.88) | 1.64(0.92~2.93) | 1.65(1.18~2.32) | 1.92(1.49~2.48) | | 1.4(0.7~2.83) | | 1.37(0.88~2.13) | | |
| [0.8, 1.5) | 1.12(1.01~1.24) | 1.26(0.98~1.62) | 0.91(0.78~1.07) | 1.33(1.18~1.49) | | 1.1(0.82~1.49) | | 1.05(0.88~1.26) | | |
| [1.5, 2.0) | 1.07(0.97~1.17) | 0.96(0.75~1.22) | 0.95(0.82~1.1) | 1.19(1.07~1.33) | | 0.92(0.69~1.23) | | 1(0.85~1.19) | | |
| [2.0, 2.5) | 1.00 (Ref.) | 1.00 (Ref.) | 1.00 (Ref.) | 1.00 (Ref.) | | 1.00 (Ref.) | | 1.00 (Ref.) | | |
| [2.5, 3.0) | 1.03(0.91~1.18) | 1.2(0.87~1.64) | 0.86(0.7~1.07) | 1.17(1~1.36) | | 1.1(0.75~1.63) | | 1.03(0.81~1.31) | | |
| [3.0, 4.0) | 1.08(0.91~1.28) | 1.48(1.02~2.15) | 1.25(0.98~1.6) | 1.19(0.98~1.44) | | 1.05(0.63~1.74) | | 1.17(0.87~1.58) | | |
| >=4.0 | 0.97(0.67~1.4) | 1.34(0.59~3.06) | 0.81(0.43~1.51) | 1.7(1.3~2.22) | | 2.27(1.26~4.09) | | 1.54(1.01~2.35) | | |
| Systemic immune-inflammation index (SII) | | |  |  | | |  | | |  |
| <300 | 1.15(0.99~1.33) | 1.03(0.69~1.52) | 0.98(0.79~1.23) | 1.22(1.05~1.41) | | 1.09(0.72~1.63) | | 1.26(1.01~1.58) | | |
| [300, 400) | 1.03(0.9~1.17) | 1.2(0.86~1.67) | 0.85(0.69~1.04) | 1.01(0.88~1.16) | | 0.94(0.64~1.38) | | 0.93(0.75~1.16) | | |
| [400, 500) | 1.00 (Ref.) | 1.00 (Ref.) | 1.00 (Ref.) | 1.00 (Ref.) | | 1.00 (Ref.) | | 1.00 (Ref.) | | |
| [500, 600) | 1.09(0.96~1.24) | 1.13(0.81~1.58) | 1(0.83~1.21) | 0.97(0.85~1.12) | | 0.75(0.5~1.13) | | 0.73(0.58~0.93) | | |
| [600, 800) | 1.18(1.05~1.33) | 1.43(1.06~1.93) | 0.94(0.79~1.12) | 1.15(1.02~1.31) | | 1.12(0.8~1.57) | | 0.99(0.82~1.21) | | |
| [800, 1500) | 1.22(1.08~1.37) | 1.48(1.1~1.99) | 0.98(0.82~1.17) | 1.18(1.04~1.34) | | 1.28(0.92~1.8) | | 1(0.82~1.23) | | |
| >=1500 | 1.56(1.32~1.86) | 2.06(1.36~3.12) | 1.46(1.13~1.9) | 1.53(1.24~1.9) | | 2.31(1.42~3.75) | | 0.94(0.63~1.41) | | |
| Neutrophil-to-lymphocyte ratio (NLR) | | |  |  | | |  | | |  |
| <1.5 | 1.00 (Ref.) | 1.00 (Ref.) | 1.00 (Ref.) | 1.00 (Ref.) | | 1.00 (Ref.) | | 1.00 (Ref.) | | |
| [1.5, 2.0) | 0.91(0.81~1.03) | 0.79(0.57~1.08) | 1.02(0.84~1.24) | 0.98(0.86~1.12) | | 0.79(0.54~1.16) | | 0.82(0.67~1.02) | | |
| [2.0, 2.5) | 1.02(0.9~1.15) | 1.06(0.78~1.43) | 1.1(0.9~1.33) | 0.95(0.83~1.09) | | 1.17(0.82~1.66) | | 0.86(0.7~1.05) | | |
| [2.5, 3.0) | 1.07(0.94~1.22) | 0.97(0.69~1.35) | 1.08(0.88~1.33) | 1.06(0.92~1.22) | | 1.1(0.75~1.62) | | 0.91(0.73~1.14) | | |
| [3.0, 3.5) | 1.13(0.98~1.31) | 0.93(0.64~1.36) | 1.15(0.92~1.45) | 1.16(0.99~1.36) | | 0.87(0.55~1.39) | | 0.94(0.73~1.21) | | |
| [3.5, 5.0) | 1.28(1.11~1.46) | 1.41(1.02~1.96) | 1.13(0.9~1.4) | 1.3(1.12~1.51) | | 1.26(0.84~1.89) | | 0.97(0.76~1.23) | | |
| >=5.0 | 1.42(1.21~1.67) | 1.88(1.3~2.73) | 1.51(1.17~1.94) | 1.51(1.25~1.83) | | 2.04(1.28~3.26) | | 1.04(0.74~1.44) | | |
| Platelet-to-lymphocyte ratio (PLR) | | |  |  | | |  | | |  |
| <80 | 1.06(0.91~1.24) | 1.35(0.94~1.94) | 1.12(0.89~1.41) | 1.4(1.19~1.63) | | 1.7(1.13~2.57) | | 1.15(0.9~1.47) | | |
| [80, 100) | 0.99(0.87~1.13) | 1.23(0.89~1.69) | 0.94(0.77~1.15) | 1.11(0.96~1.29) | | 1.12(0.74~1.69) | | 0.98(0.78~1.22) | | |
| [100, 120) | 1.00 (Ref.) | 1.00 (Ref.) | 1.00 (Ref.) | 1.00 (Ref.) | | 1.00 (Ref.) | | 1.00 (Ref.) | | |
| [120, 150) | 1.02(0.91~1.14) | 1.12(0.84~1.5) | 1.04(0.88~1.23) | 1.09(0.96~1.23) | | 1.35(0.95~1.91) | | 0.87(0.72~1.06) | | |
| [150, 200) | 1.04(0.93~1.16) | 1.02(0.76~1.38) | 0.9(0.75~1.07) | 1.16(1.03~1.32) | | 1.05(0.73~1.53) | | 0.92(0.75~1.12) | | |
| [200, 250) | 1.13(0.98~1.3) | 1.58(1.12~2.23) | 0.95(0.75~1.19) | 1.25(1.07~1.47) | | 1.65(1.08~2.52) | | 0.99(0.77~1.28) | | |
| >=250 | 1.13(0.97~1.33) | 1.64(1.12~2.4) | 1.29(1.02~1.64) | 1.44(1.2~1.72) | | 2.05(1.3~3.23) | | 1.04(0.77~1.41) | | |
| Lymphocyte-to-monocyte ratio (LMR) | | |  |  | | |  | | |  |
| <2.5 | 1.39(1.2~1.61) | 1.8(1.23~2.65) | 1.43(1.12~1.83) | 1.48(1.25~1.75) | | 2.65(1.52~4.64) | | 0.94(0.72~1.21) | | |
| [2.5, 3.0) | 1.08(0.92~1.26) | 1.27(0.84~1.91) | 1.16(0.89~1.5) | 1.14(0.96~1.37) | | 1.91(1.06~3.42) | | 0.71(0.54~0.94) | | |
| [3.0, 4.0) | 1.04(0.91~1.2) | 1.11(0.76~1.6) | 1.15(0.92~1.45) | 1.11(0.95~1.3) | | 2.24(1.31~3.81) | | 0.84(0.66~1.06) | | |
| [4.0, 5.0) | 0.98(0.85~1.13) | 1.24(0.86~1.8) | 1.07(0.84~1.36) | 1.05(0.9~1.24) | | 1.84(1.07~3.18) | | 0.74(0.58~0.95) | | |
| [5.0, 6.0) | 0.94(0.81~1.1) | 0.99(0.65~1.51) | 1.08(0.83~1.4) | 1.04(0.87~1.24) | | 1.89(1.06~3.37) | | 0.74(0.57~0.97) | | |
| [6.0, 8.0) | 1.00 (Ref.) | 1.00 (Ref.) | 1.00 (Ref.) | 1.00 (Ref.) | | 1.00 (Ref.) | | 1.00 (Ref.) | | |
| >=8.0 | 1.02(0.83~1.27) | 1.4(0.84~2.33) | 1.42(1.02~1.97) | 1.25(1.01~1.55) | | 1.86(0.93~3.72) | | 0.95(0.68~1.32) | | |
| Ref., Reference.  a Adjusted age, sex, race, townsend deprivation index, education, BMI, smoking status, frequency of alcohol drinking, physical activity, hypertension, heart attack, angina, stroke, diabetes, hyperlipidemia, diseases of blood and blood-forming organs, and malignant neoplasms.  b Adjusted age, sex, race, townsend deprivation index, education, BMI, smoking status, frequency of alcohol drinking, physical activity, hypertension, heart attack, angina, stroke, diabetes, hyperlipidemia, diseases of blood and blood-forming organs, and chronic diseases involving the immune mechanism. | | | | | | | | | | |

**Table S6.** The hazard risk (HR) with 95% confidence intervals (95%CI) between various systematic information indicators and three arrhythmia subtypes stratified by age group at baseline using the Cox proportional hazard model.

| Variables | Atrial Fibrillation/Flutter | | Ventricular Arrhythmia | | Bradyarrhythmia | | |  |
| --- | --- | --- | --- | --- | --- | --- | --- | --- |
|  | **Age at baseline**  **<60** | **Age at baseline**  **>=60** | **Age at baseline**  **<60** | **Age at baseline**  **>=60** | **Age at baseline**  **<60** | **Age at baseline**  **>=60** |  |  |
|  | **HR (95%CI)** | **HR (95%CI)** | **HR (95%CI)** | **HR (95%CI)** | **HR (95%CI)** | **HR (95%CI)** |  |  |
| C-reactive protein (mg/L) | **P of interaction = 0.0763** | | **P of interaction = 0.305** | | **P of interaction = 0.0006** | | |  |
| <0.5 | 1.00 (Ref.) | 1.00 (Ref.) | 1.00 (Ref.) | 1.00 (Ref.) | 1.00 (Ref.) | 1.00 (Ref.) |  |  |
| [0.5, 1.0) | 0.98(0.9~1.08) | 1(0.95~1.07) | 1.22(1~1.5) | 0.95(0.8~1.12) | 1.15(1~1.32) | 0.99(0.91~1.09) |  |  |
| [1.0, 2.0) | 0.97(0.89~1.06) | 1.01(0.95~1.07) | 1.36(1.11~1.66) | 1.1(0.94~1.29) | 1.1(0.96~1.26) | 0.99(0.9~1.08) |  |  |
| [2.0, 3.0) | 1.02(0.92~1.13) | 1.04(0.98~1.11) | 1.24(0.98~1.57) | 1.05(0.87~1.26) | 1.16(1~1.36) | 1.02(0.92~1.12) |  |  |
| [3.0, 4.0) | 1.08(0.95~1.21) | 1.05(0.97~1.13) | 1.4(1.06~1.83) | 1.2(0.97~1.47) | 1.31(1.09~1.57) | 1.08(0.96~1.21) |  |  |
| [4.0, 10.0) | 1.07(0.96~1.19) | 1.16(1.08~1.24) | 1.5(1.18~1.91) | 1.39(1.16~1.66) | 1.29(1.1~1.52) | 1.12(1.01~1.24) |  |  |
| >=10.0 | 1.35(1.18~1.54) | 1.31(1.21~1.43) | 2.28(1.72~3.02) | 1.63(1.31~2.03) | 1.7(1.39~2.08) | 1.14(1~1.31) |  |  |
| Neutrophil count (10^9 cells/L) | **P of interaction = 0.1155** | | **P of interaction = 0.3223** | | **P of interaction = 0.1111** | | |  |
| <2.0 | 1.00 (Ref.) | 1.00 (Ref.) | 1.00 (Ref.) | 1.00 (Ref.) | 1.00 (Ref.) | 1.00 (Ref.) |  |  |
| [2.0, 3.0) | 0.92(0.75~1.13) | 1.07(0.92~1.25) | 0.84(0.55~1.29) | 1.53(0.91~2.59) | 0.89(0.66~1.19) | 0.82(0.66~1.01) |  |  |
| [3.0, 4.0) | 0.92(0.75~1.12) | 1.02(0.88~1.19) | 0.87(0.58~1.32) | 1.58(0.94~2.64) | 0.95(0.71~1.27) | 0.84(0.68~1.03) |  |  |
| [4.0, 5.0) | 0.96(0.79~1.18) | 1.07(0.92~1.24) | 0.89(0.59~1.35) | 1.62(0.97~2.71) | 0.96(0.72~1.28) | 0.86(0.7~1.05) |  |  |
| [5.0, 6.0) | 0.94(0.77~1.16) | 1.14(0.98~1.33) | 1.01(0.66~1.53) | 2.07(1.24~3.47) | 1.06(0.79~1.42) | 0.88(0.72~1.09) |  |  |
| [6.0, 7.5) | 1(0.81~1.24) | 1.25(1.07~1.47) | 1.31(0.85~2.02) | 2.08(1.23~3.51) | 1.08(0.79~1.47) | 0.92(0.74~1.15) |  |  |
| >=7.5 | 1.34(1.06~1.69) | 1.39(1.17~1.64) | 1.66(1.04~2.64) | 2.95(1.72~5.07) | 1.26(0.89~1.77) | 0.96(0.75~1.22) |  |  |
| Monocyte count (10^9 cells/L) | **P of interaction = 0.0183** | | **P of interaction = 0.3825** | | **P of interaction = 0.0013** | | |  |
| <0.3 | 1.00 (Ref.) | 1.00 (Ref.) | 1.00 (Ref.) | 1.00 (Ref.) | 1.00 (Ref.) | 1.00 (Ref.) |  |  |
| [0.3, 0.4) | 1.01(0.91~1.13) | 0.99(0.91~1.06) | 0.85(0.67~1.09) | 1(0.8~1.26) | 1.01(0.85~1.2) | 0.94(0.84~1.06) |  |  |
| [0.4, 0.5) | 0.95(0.85~1.06) | 1.02(0.95~1.09) | 0.84(0.67~1.06) | 1.17(0.95~1.45) | 0.96(0.81~1.13) | 0.97(0.87~1.08) |  |  |
| [0.5, 0.6) | 0.99(0.89~1.11) | 1.02(0.95~1.1) | 0.96(0.76~1.22) | 1.2(0.97~1.48) | 1.07(0.9~1.26) | 0.95(0.85~1.06) |  |  |
| [0.6, 0.7) | 1(0.89~1.13) | 1.1(1.02~1.19) | 0.87(0.67~1.13) | 1.21(0.97~1.52) | 1.01(0.84~1.21) | 1.02(0.91~1.14) |  |  |
| [0.7, 0.8) | 1.13(0.99~1.3) | 1.14(1.04~1.24) | 1.18(0.89~1.57) | 1.38(1.08~1.75) | 1.22(0.99~1.49) | 1.07(0.94~1.22) |  |  |
| >=0.8 | 1.14(0.99~1.31) | 1.2(1.1~1.31) | 1.43(1.08~1.89) | 1.74(1.38~2.2) | 1.1(0.89~1.36) | 1(0.88~1.15) |  |  |
| Lymphocyte count (10^9 cells/L) | **P of interaction = 0.076** | | **P of interaction = 0.5952** | | **P of interaction = 0.0213** | | |  |
| <0.8 | 1.8(1.4~2.32) | 1.5(1.3~1.74) | 1.72(1~2.96) | 1.25(0.81~1.92) | 1.07(0.67~1.71) | 1.41(1.12~1.78) |  |  |
| [0.8, 1.5) | 1.19(1.1~1.28) | 1.12(1.07~1.17) | 1.23(1.05~1.45) | 1.13(1~1.28) | 1.03(0.91~1.15) | 1.04(0.97~1.12) |  |  |
| [1.5, 2.0) | 1.1(1.03~1.18) | 1.04(0.99~1.08) | 0.92(0.79~1.07) | 1(0.89~1.12) | 1.07(0.97~1.18) | 1.03(0.96~1.09) |  |  |
| [2.0, 2.5) | 1.00 (Ref.) | 1.00 (Ref.) | 1.00 (Ref.) | 1.00 (Ref.) | 1.00 (Ref.) | 1.00 (Ref.) |  |  |
| [2.5, 3.0) | 0.93(0.85~1.03) | 0.98(0.93~1.04) | 1(0.82~1.22) | 0.99(0.85~1.16) | 0.98(0.85~1.12) | 0.93(0.84~1.01) |  |  |
| [3.0, 4.0) | 1.05(0.93~1.19) | 0.96(0.89~1.04) | 1.04(0.8~1.35) | 1.1(0.9~1.34) | 1.37(1.16~1.61) | 1.09(0.97~1.22) |  |  |
| >=4.0 | 1.32(1.01~1.74) | 1.04(0.88~1.22) | 1.12(0.61~2.06) | 1.35(0.92~2) | 1.21(0.8~1.84) | 1.07(0.83~1.38) |  |  |
| Systemic immune-inflammation index (SII) | **P of interaction = 0.0928** | | **P of interaction = 0.3004** | | **P of interaction = 0.1291** | | |  |
| <300 | 1.14(1.03~1.27) | 1.04(0.98~1.11) | 1.19(0.95~1.49) | 1.11(0.93~1.32) | 1.18(1.02~1.37) | 1.08(0.98~1.18) |  |  |
| [300, 400) | 1.17(1.07~1.28) | 1.02(0.96~1.07) | 0.98(0.79~1.21) | 1.21(1.04~1.42) | 1.13(0.98~1.29) | 0.96(0.88~1.04) |  |  |
| [400, 500) | 1.00 (Ref.) | 1.00 (Ref.) | 1.00 (Ref.) | 1.00 (Ref.) | 1.00 (Ref.) | 1.00 (Ref.) |  |  |
| [500, 600) | 1.14(1.04~1.25) | 0.99(0.94~1.05) | 1.11(0.91~1.36) | 1.15(0.99~1.34) | 1.08(0.95~1.24) | 0.99(0.91~1.08) |  |  |
| [600, 800) | 1.13(1.04~1.23) | 1.05(1~1.1) | 1.15(0.95~1.39) | 1.16(1.01~1.35) | 1.16(1.02~1.32) | 1.01(0.93~1.09) |  |  |
| [800, 1500) | 1.25(1.14~1.36) | 1.1(1.04~1.16) | 1.46(1.2~1.77) | 1.38(1.19~1.6) | 1.11(0.97~1.27) | 1.07(0.99~1.16) |  |  |
| >=1500 | 1.57(1.3~1.88) | 1.46(1.32~1.61) | 2.72(1.97~3.75) | 2.26(1.77~2.87) | 1.72(1.32~2.24) | 1.16(0.99~1.38) |  |  |
| Neutrophil-to-lymphocyte ratio (NLR) | **P of interaction = 0.5934** | | **P of interaction = 0.0649** | | **P of interaction = 0.9475** | | |  |
| <1.5 | 1.00 (Ref.) | 1.00 (Ref.) | 1.00 (Ref.) | 1.00 (Ref.) | 1.00 (Ref.) | 1.00 (Ref.) |  |  |
| [1.5, 2.0) | 0.97(0.89~1.06) | 1(0.95~1.06) | 0.96(0.79~1.16) | 1.05(0.9~1.22) | 0.93(0.82~1.05) | 1(0.92~1.09) |  |  |
| [2.0, 2.5) | 1.01(0.92~1.1) | 1.06(1.01~1.12) | 0.97(0.8~1.18) | 1.18(1.02~1.38) | 0.98(0.86~1.11) | 1.03(0.95~1.12) |  |  |
| [2.5, 3.0) | 1.1(1~1.21) | 1.1(1.04~1.16) | 1.14(0.93~1.4) | 1.08(0.91~1.28) | 0.99(0.86~1.14) | 1.03(0.94~1.13) |  |  |
| [3.0, 3.5) | 1.18(1.06~1.31) | 1.16(1.09~1.24) | 1.37(1.09~1.72) | 1.15(0.96~1.39) | 1.08(0.93~1.27) | 1.14(1.03~1.25) |  |  |
| [3.5, 5.0) | 1.31(1.18~1.46) | 1.28(1.2~1.36) | 1.51(1.21~1.89) | 1.49(1.25~1.77) | 1.06(0.9~1.24) | 1.12(1.02~1.24) |  |  |
| >=5.0 | 1.52(1.3~1.79) | 1.46(1.34~1.6) | 2.13(1.57~2.9) | 1.84(1.47~2.3) | 1.22(0.95~1.56) | 1.25(1.08~1.43) |  |  |
| Platelet-to-lymphocyte ratio (PLR) | **P of interaction = 0.0849** | | **P of interaction = 0.6489** | | **P of interaction = 0.0171** | | |  |
| <80 | 1.08(0.97~1.21) | 1.08(1.01~1.15) | 1.22(0.97~1.54) | 1.11(0.94~1.32) | 1.21(1.04~1.41) | 1.06(0.97~1.17) |  |  |
| [80, 100) | 1.01(0.92~1.11) | 1.05(0.99~1.11) | 1.13(0.92~1.38) | 1.08(0.93~1.26) | 1.03(0.9~1.17) | 1(0.92~1.08) |  |  |
| [100, 120) | 1.00 (Ref.) | 1.00 (Ref.) | 1.00 (Ref.) | 1.00 (Ref.) | 1.00 (Ref.) | 1.00 (Ref.) |  |  |
| [120, 150) | 1.07(0.99~1.16) | 1(0.95~1.05) | 1.06(0.89~1.27) | 1.03(0.9~1.18) | 0.97(0.87~1.1) | 0.99(0.92~1.06) |  |  |
| [150, 200) | 1.02(0.94~1.11) | 1.05(0.99~1.1) | 1.01(0.84~1.22) | 1.01(0.88~1.17) | 0.98(0.87~1.11) | 0.98(0.91~1.05) |  |  |
| [200, 250) | 1.07(0.96~1.2) | 1.03(0.96~1.11) | 1.41(1.11~1.78) | 1.15(0.95~1.38) | 0.97(0.81~1.15) | 1.03(0.93~1.15) |  |  |
| >=250 | 1.29(1.12~1.48) | 1.15(1.06~1.26) | 1.69(1.27~2.25) | 1.36(1.08~1.7) | 1.11(0.89~1.38) | 1.11(0.97~1.27) |  |  |
| Lymphocyte-to-monocyte ratio (LMR) | **P of interaction = 0.0043** | | **P of interaction = 0.002** | | **P of interaction = 0.395** | | |  |
| <2.5 | 1.47(1.31~1.65) | 1.37(1.28~1.47) | 1.45(1.13~1.84) | 1.95(1.57~2.42) | 1.18(0.99~1.4) | 1.16(1.04~1.29) |  |  |
| [2.5, 3.0) | 1.19(1.06~1.34) | 1.2(1.11~1.29) | 1.46(1.15~1.84) | 1.74(1.39~2.17) | 0.97(0.81~1.15) | 1.01(0.91~1.13) |  |  |
| [3.0, 4.0) | 1.14(1.03~1.26) | 1.12(1.05~1.19) | 0.95(0.77~1.17) | 1.54(1.25~1.89) | 1.04(0.9~1.21) | 1.02(0.93~1.13) |  |  |
| [4.0, 5.0) | 1.06(0.96~1.18) | 1.07(1~1.14) | 0.94(0.76~1.17) | 1.46(1.18~1.8) | 0.98(0.85~1.14) | 0.97(0.88~1.07) |  |  |
| [5.0, 6.0) | 1.06(0.95~1.18) | 1.04(0.97~1.11) | 0.81(0.64~1.03) | 1.36(1.09~1.71) | 0.93(0.79~1.1) | 0.97(0.87~1.08) |  |  |
| [6.0, 8.0) | 1.00 (Ref.) | 1.00 (Ref.) | 1.00 (Ref.) | 1.00 (Ref.) | 1.00 (Ref.) | 1.00 (Ref.) |  |  |
| >=8.0 | 1.1(0.95~1.28) | 0.98(0.89~1.09) | 1.09(0.8~1.48) | 1.5(1.12~2.02) | 1.17(0.95~1.44) | 1.03(0.88~1.2) |  |  |
| Ref., Reference.  Adjusted age, sex, race, townsend deprivation index, education, BMI, smoking status, frequency of alcohol drinking, and physical activity, hypertension, heart attack, angina, stroke, diabetes, hyperlipidemia, diseases of blood and blood-forming organs, chronic diseases involving the immune mechanism, and malignant neoplasms. | | | | | | | | |

**Table S7.** The hazard risk (HR) with 95% confidence intervals (95%CI) between various systematic information indicators and three arrhythmia subtypes stratified by sex using the Cox proportional hazard model.

| Variables | Atrial Fibrillation/Flutter | | Ventricular Arrhythmia | | | | Bradyarrhythmia | | | | | |
| --- | --- | --- | --- | --- | --- | --- | --- | --- | --- | --- | --- | --- |
|  | **Female** | **Male** | **Female** | | **Male** | **Female** | | **Male** |  |  |  |  |
|  | **HR (95%CI)** | **HR (95%CI)** | **HR (95%CI)** | | **HR (95%CI)** | **HR (95%CI)** | | **HR (95%CI)** |  |  |  |  |
| C-reactive protein (mg/L) | **P of interaction = 0.3853** | | | **P of interaction = 0.0301** | | | **P of interaction = 0.8838** | | | |  |  |
| <0.5 | 1.00 (Ref.) | 1.00 (Ref.) | 1.00 (Ref.) | | 1.00 (Ref.) | 1.00 (Ref.) | | 1.00 (Ref.) |  |  |  |  |
| [0.5, 1.0) | 1.02(0.93~1.11) | 0.99(0.93~1.05) | 1.17(0.9~1.51) | | 1.01(0.87~1.18) | 1.02(0.89~1.18) | | 1.05(0.96~1.15) |  |  |  |  |
| [1.0, 2.0) | 1.01(0.93~1.1) | 0.99(0.93~1.05) | 1.54(1.21~1.96) | | 1.09(0.95~1.27) | 0.99(0.86~1.13) | | 1.04(0.96~1.14) |  |  |  |  |
| [2.0, 3.0) | 1.07(0.97~1.17) | 1.02(0.95~1.09) | 1.35(1.03~1.78) | | 1.05(0.89~1.25) | 1.06(0.91~1.24) | | 1.07(0.97~1.18) |  |  |  |  |
| [3.0, 4.0) | 1.12(1.01~1.25) | 1.02(0.94~1.1) | 1.34(0.98~1.84) | | 1.28(1.05~1.55) | 1.11(0.93~1.32) | | 1.17(1.05~1.32) |  |  |  |  |
| [4.0, 10.0) | 1.13(1.03~1.25) | 1.14(1.07~1.23) | 1.57(1.19~2.06) | | 1.43(1.21~1.7) | 1.17(1~1.37) | | 1.19(1.07~1.32) |  |  |  |  |
| >=10.0 | 1.37(1.22~1.54) | 1.31(1.19~1.43) | 2.56(1.89~3.49) | | 1.59(1.29~1.98) | 1.35(1.12~1.64) | | 1.26(1.1~1.45) |  |  |  |  |
| Neutrophil count (10^9 cells/L) | **P of interaction = 0.5889** | | | **P of interaction = 0.3405** | | | **P of interaction = 0.1442** | | | |  |  |
| <2.0 | 1.00 (Ref.) | 1.00 (Ref.) | 1.00 (Ref.) | | 1.00 (Ref.) | 1.00 (Ref.) | | 1.00 (Ref.) |  |  |  |  |
| [2.0, 3.0) | 1.09(0.89~1.33) | 0.98(0.84~1.14) | 1.28(0.69~2.38) | | 1.04(0.7~1.52) | 0.84(0.62~1.13) | | 0.85(0.68~1.05) |  |  |  |  |
| [3.0, 4.0) | 1.07(0.88~1.3) | 0.94(0.8~1.09) | 1.4(0.77~2.56) | | 1.04(0.71~1.51) | 0.85(0.64~1.14) | | 0.89(0.73~1.09) |  |  |  |  |
| [4.0, 5.0) | 1.11(0.91~1.35) | 0.99(0.85~1.15) | 1.28(0.7~2.35) | | 1.11(0.77~1.62) | 0.85(0.63~1.13) | | 0.92(0.75~1.13) |  |  |  |  |
| [5.0, 6.0) | 1.12(0.91~1.37) | 1.05(0.9~1.23) | 1.76(0.95~3.24) | | 1.3(0.89~1.9) | 1(0.74~1.34) | | 0.92(0.75~1.14) |  |  |  |  |
| [6.0, 7.5) | 1.26(1.03~1.56) | 1.13(0.96~1.32) | 1.88(1.01~3.51) | | 1.45(0.99~2.14) | 1.01(0.74~1.38) | | 0.98(0.79~1.21) |  |  |  |  |
| >=7.5 | 1.42(1.13~1.79) | 1.34(1.13~1.58) | 2.73(1.42~5.24) | | 1.91(1.27~2.88) | 1.12(0.79~1.6) | | 1.03(0.81~1.32) |  |  |  |  |
| Monocyte count (10^9 cells/L) | **P of interaction = 0.0116** | | | **P of interaction = 0.4851** | | | **P of interaction = 0.0131** | | | |  |  |
| <0.3 | 1.00 (Ref.) | 1.00 (Ref.) | 1.00 (Ref.) | | 1.00 (Ref.) | 1.00 (Ref.) | | 1.00 (Ref.) |  |  |  |  |
| [0.3, 0.4) | 1.03(0.95~1.12) | 0.95(0.86~1.04) | 0.98(0.77~1.25) | | 0.88(0.7~1.1) | 0.95(0.82~1.09) | | 0.98(0.86~1.11) |  |  |  |  |
| [0.4, 0.5) | 1.05(0.96~1.14) | 0.93(0.85~1.01) | 1.08(0.86~1.37) | | 0.94(0.76~1.16) | 0.94(0.82~1.07) | | 0.97(0.86~1.09) |  |  |  |  |
| [0.5, 0.6) | 1.05(0.96~1.15) | 0.95(0.87~1.04) | 1.23(0.97~1.57) | | 0.97(0.79~1.2) | 1.07(0.93~1.24) | | 0.93(0.83~1.06) |  |  |  |  |
| [0.6, 0.7) | 1.17(1.06~1.29) | 0.99(0.91~1.09) | 1.11(0.83~1.47) | | 0.98(0.79~1.22) | 1.05(0.89~1.23) | | 1(0.88~1.13) |  |  |  |  |
| [0.7, 0.8) | 1.24(1.1~1.4) | 1.05(0.96~1.16) | 1.33(0.95~1.88) | | 1.18(0.94~1.48) | 1.27(1.04~1.54) | | 1.06(0.93~1.22) |  |  |  |  |
| >=0.8 | 1.25(1.09~1.43) | 1.12(1.02~1.23) | 1.93(1.38~2.69) | | 1.44(1.15~1.8) | 1.14(0.91~1.43) | | 1(0.87~1.15) |  |  |  |  |
| Lymphocyte count (10^9 cells/L) | **P of interaction = 0.6037** | | | **P of interaction = 0.8564** | | | **P of interaction = 0.4585** | | | |  |  |
| <0.8 | 1.4(1.08~1.8) | 1.65(1.42~1.91) | 1.12(0.5~2.53) | | 1.45(1~2.1) | 1.14(0.72~1.83) | | 1.42(1.13~1.79) |  |  |  |  |
| [0.8, 1.5) | 1.14(1.06~1.21) | 1.14(1.08~1.19) | 1.27(1.06~1.53) | | 1.12(1~1.27) | 1.04(0.93~1.17) | | 1.05(0.98~1.13) |  |  |  |  |
| [1.5, 2.0) | 1.03(0.98~1.09) | 1.07(1.02~1.12) | 0.99(0.84~1.17) | | 0.96(0.86~1.07) | 0.99(0.9~1.09) | | 1.06(0.99~1.13) |  |  |  |  |
| [2.0, 2.5) | 1.00 (Ref.) | 1.00 (Ref.) | 1.00 (Ref.) | | 1.00 (Ref.) | 1.00 (Ref.) | | 1.00 (Ref.) |  |  |  |  |
| [2.5, 3.0) | 0.97(0.9~1.05) | 0.96(0.9~1.02) | 1.06(0.86~1.3) | | 0.95(0.81~1.11) | 0.86(0.76~0.98) | | 0.98(0.89~1.08) |  |  |  |  |
| [3.0, 4.0) | 0.93(0.84~1.02) | 1.04(0.95~1.13) | 0.98(0.75~1.27) | | 1.12(0.92~1.36) | 1.15(0.99~1.33) | | 1.18(1.04~1.33) |  |  |  |  |
| >=4.0 | 1.09(0.88~1.34) | 1.09(0.9~1.32) | 1.24(0.72~2.14) | | 1.26(0.84~1.9) | 0.86(0.58~1.28) | | 1.25(0.97~1.63) |  |  |  |  |
| Systemic immune-inflammation index (SII) | **P of interaction = 0.3689** | | | **P of interaction = 0.084** | | | **P of interaction = 0.0098** | | | |  |  |
| <300 | 1.09(1~1.19) | 1.05(0.99~1.12) | 1.35(1.05~1.74) | | 1.06(0.9~1.25) | 1.13(0.98~1.32) | | 1.09(0.99~1.19) |  |  |  |  |
| [300, 400) | 1.04(0.96~1.12) | 1.06(1~1.13) | 0.99(0.78~1.25) | | 1.18(1.02~1.37) | 1.01(0.88~1.15) | | 1(0.92~1.08) |  |  |  |  |
| [400, 500) | 1.00 (Ref.) | 1.00 (Ref.) | 1.00 (Ref.) | | 1.00 (Ref.) | 1.00 (Ref.) | | 1.00 (Ref.) |  |  |  |  |
| [500, 600) | 1.07(0.99~1.15) | 1.01(0.95~1.07) | 1.08(0.86~1.36) | | 1.16(1~1.35) | 1.1(0.97~1.25) | | 0.98(0.9~1.07) |  |  |  |  |
| [600, 800) | 1.06(0.99~1.14) | 1.07(1.02~1.14) | 1.23(1~1.52) | | 1.12(0.98~1.29) | 1.06(0.94~1.2) | | 1.05(0.97~1.13) |  |  |  |  |
| [800, 1500) | 1.09(1.01~1.17) | 1.17(1.11~1.24) | 1.52(1.23~1.87) | | 1.36(1.19~1.57) | 1.2(1.06~1.36) | | 1.04(0.96~1.13) |  |  |  |  |
| >=1500 | 1.48(1.27~1.71) | 1.5(1.35~1.67) | 2.56(1.81~3.63) | | 2.32(1.84~2.92) | 1.75(1.39~2.22) | | 1.13(0.95~1.34) |  |  |  |  |
| Neutrophil-to-lymphocyte ratio (NLR) | **P of interaction = 0.1752** | | **P of interaction = 0.0152** | | | | **P of interaction = 0.080** | | |  |  |  |
| <1.5 | 1.00 (Ref.) | 1.00 (Ref.) | 1.00 (Ref.) | | 1.00 (Ref.) | 1.00 (Ref.) | | 1.00 (Ref.) |  |  |  |  |
| [1.5, 2.0) | 1.03(0.96~1.1) | 0.97(0.91~1.03) | 1.04(0.85~1.26) | | 1(0.85~1.16) | 0.95(0.84~1.06) | | 1(0.91~1.09) |  |  |  |  |
| [2.0, 2.5) | 1.12(1.04~1.2) | 1(0.95~1.07) | 0.99(0.8~1.21) | | 1.15(0.99~1.34) | 1.04(0.92~1.16) | | 1(0.92~1.09) |  |  |  |  |
| [2.5, 3.0) | 1.12(1.03~1.21) | 1.09(1.02~1.16) | 1.05(0.84~1.32) | | 1.12(0.95~1.31) | 1.04(0.91~1.18) | | 1.02(0.93~1.11) |  |  |  |  |
| [3.0, 3.5) | 1.19(1.08~1.31) | 1.15(1.07~1.23) | 1.29(0.99~1.67) | | 1.2(1.01~1.43) | 1.18(1.01~1.38) | | 1.1(1~1.22) |  |  |  |  |
| [3.5, 5.0) | 1.3(1.18~1.43) | 1.27(1.19~1.36) | 1.74(1.36~2.23) | | 1.42(1.2~1.68) | 1.09(0.93~1.29) | | 1.11(1.01~1.23) |  |  |  |  |
| >=5.0 | 1.6(1.38~1.85) | 1.44(1.31~1.58) | 2.65(1.9~3.7) | | 1.73(1.39~2.15) | 1.59(1.25~2.02) | | 1.17(1.02~1.35) |  |  |  |  |
| Platelet-to-lymphocyte ratio (PLR) | **P of interaction = 0.2644** | | **P of interaction = 0.9168** | | | | **P of interaction = 0.5161** | | |  |  |  |
| <80 | 1.13(1.03~1.24) | 1.06(0.99~1.13) | 1.04(0.78~1.37) | | 1.18(1.01~1.38) | 1.08(0.92~1.27) | | 1.1(1~1.21) |  |  |  |  |
| [80, 100) | 1.01(0.94~1.1) | 1.06(1~1.12) | 1.09(0.87~1.36) | | 1.1(0.95~1.27) | 0.99(0.87~1.14) | | 1.01(0.93~1.1) |  |  |  |  |
| [100, 120) | 1.00 (Ref.) | 1.00 (Ref.) | 1.00 (Ref.) | | 1.00 (Ref.) | 1.00 (Ref.) | | 1.00 (Ref.) |  |  |  |  |
| [120, 150) | 1(0.93~1.07) | 1.03(0.98~1.08) | 1.05(0.87~1.28) | | 1.04(0.91~1.18) | 1.02(0.91~1.15) | | 0.97(0.9~1.04) |  |  |  |  |
| [150, 200) | 1.03(0.96~1.11) | 1.04(0.99~1.1) | 1.04(0.85~1.27) | | 1(0.87~1.15) | 1.05(0.94~1.18) | | 0.95(0.88~1.03) |  |  |  |  |
| [200, 250) | 1.02(0.93~1.12) | 1.06(0.99~1.14) | 1.28(0.99~1.65) | | 1.22(1.02~1.46) | 1.01(0.85~1.18) | | 1.02(0.92~1.14) |  |  |  |  |
| >=250 | 1.12(0.99~1.26) | 1.24(1.13~1.36) | 1.71(1.27~2.31) | | 1.37(1.1~1.7) | 1.27(1.04~1.55) | | 1.05(0.91~1.21) |  |  |  |  |
| Lymphocyte-to-monocyte ratio (LMR) | **P of interaction = 0.0001** | | **P of interaction = 0.054** | | | | **P of interaction = 0.0144** | | |  |  |  |
| <2.5 | 1.38(1.24~1.53) | 1.39(1.28~1.52) | 2.17(1.65~2.87) | | 1.59(1.29~1.97) | 1.44(1.21~1.71) | | 1.09(0.97~1.23) |  |  |  |  |
| [2.5, 3.0) | 1.36(1.23~1.49) | 1.15(1.05~1.25) | 1.51(1.14~2) | | 1.57(1.26~1.95) | 1.09(0.92~1.3) | | 0.95(0.85~1.08) |  |  |  |  |
| [3.0, 4.0) | 1.14(1.06~1.23) | 1.11(1.02~1.2) | 1.25(1.01~1.56) | | 1.24(1.01~1.52) | 1.08(0.96~1.23) | | 0.98(0.88~1.1) |  |  |  |  |
| [4.0, 5.0) | 1.05(0.97~1.13) | 1.07(0.98~1.17) | 1.23(1~1.52) | | 1.17(0.95~1.45) | 0.99(0.87~1.12) | | 0.94(0.84~1.06) |  |  |  |  |
| [5.0, 6.0) | 1.04(0.96~1.13) | 1.04(0.95~1.14) | 1.03(0.81~1.29) | | 1.12(0.89~1.42) | 0.95(0.83~1.08) | | 0.95(0.83~1.08) |  |  |  |  |
| [6.0, 8.0) | 1.00 (Ref.) | 1.00 (Ref.) | 1.00 (Ref.) | | 1.00 (Ref.) | 1.00 (Ref.) | | 1.00 (Ref.) |  |  |  |  |
| >=8.0 | 0.94(0.84~1.05) | 1.15(1~1.32) | 1.07(0.79~1.44) | | 1.56(1.15~2.12) | 1.05(0.89~1.25) | | 1.1(0.92~1.32) |  |  |  |  |
| Ref., Reference.  Adjusted age, race, townsend deprivation index, education, BMI, smoking status, frequency of alcohol drinking, and physical activity, hypertension, heart attack, angina, stroke, diabetes, hyperlipidemia, diseases of blood and blood-forming organs, chronic diseases involving the immune mechanism, and malignant neoplasms. | | | | | | | | | | | |  |

**Table S8.** The hazard risk (HR) with 95% confidence intervals (95%CI) between various systematic information indicators and three arrhythmia subtypes excluding incident cases occurred in the first 2 years of follow-up (N=4833) by the Cox proportional hazard model.

| Variables | Atrial Fibrillation/Flutter | Ventricular Arrhythmia | Bradyarrhythmia |
| --- | --- | --- | --- |
|  | **HR (95%CI)** | **HR (95%CI)** | **HR (95%CI)** |
| C-reactive protein (mg/L) |  |  |  |
| <0.5 | 1.00 (Ref.) | 1.00 (Ref.) | 1.00 (Ref.) |
| [0.5, 1.0) | 1.01(0.95~1.06) | 1.07(0.94~1.23) | 1.05(0.98~1.14) |
| [1.0, 2.0) | 1(0.95~1.05) | 1.22(1.07~1.39) | 1.03(0.95~1.11) |
| [2.0, 3.0) | 1.04(0.98~1.1) | 1.14(0.98~1.33) | 1.06(0.98~1.16) |
| [3.0, 4.0) | 1.04(0.98~1.11) | 1.27(1.06~1.51) | 1.14(1.03~1.26) |
| [4.0, 10.0) | 1.13(1.06~1.2) | 1.44(1.24~1.68) | 1.18(1.08~1.29) |
| >=10.0 | 1.32(1.22~1.42) | 1.79(1.48~2.15) | 1.27(1.13~1.42) |
| Neutrophil count (10^9 cells/L) |  |  |  |
| <2.0 | 1.00 (Ref.) | 1.00 (Ref.) | 1.00 (Ref.) |
| [2.0, 3.0) | 1.03(0.9~1.17) | 1.15(0.81~1.63) | 0.84(0.71~1.01) |
| [3.0, 4.0) | 0.99(0.87~1.12) | 1.18(0.84~1.65) | 0.88(0.74~1.04) |
| [4.0, 5.0) | 1.03(0.91~1.17) | 1.21(0.86~1.7) | 0.9(0.76~1.07) |
| [5.0, 6.0) | 1.07(0.94~1.22) | 1.45(1.03~2.05) | 0.93(0.78~1.1) |
| [6.0, 7.5) | 1.17(1.03~1.34) | 1.67(1.17~2.37) | 0.97(0.81~1.16) |
| >=7.5 | 1.34(1.16~1.55) | 2.14(1.48~3.1) | 1.02(0.83~1.26) |
| Monocyte count (10^9 cells/L) |  |  |  |
| <0.3 | 1.00 (Ref.) | 1.00 (Ref.) | 1.00 (Ref.) |
| [0.3, 0.4) | 1(0.93~1.06) | 0.96(0.8~1.14) | 0.98(0.89~1.08) |
| [0.4, 0.5) | 1(0.94~1.07) | 1.05(0.89~1.24) | 0.98(0.9~1.08) |
| [0.5, 0.6) | 1.02(0.95~1.08) | 1.13(0.95~1.33) | 1.01(0.91~1.11) |
| [0.6, 0.7) | 1.08(1.01~1.15) | 1.09(0.91~1.3) | 1.04(0.94~1.16) |
| [0.7, 0.8) | 1.12(1.04~1.21) | 1.32(1.09~1.59) | 1.13(1.01~1.26) |
| >=0.8 | 1.17(1.09~1.27) | 1.62(1.34~1.96) | 1.02(0.91~1.15) |
| Lymphocyte count (10^9 cells/L) |  |  |  |
| <0.8 | 1.59(1.39~1.81) | 1.38(0.96~1.96) | 1.35(1.09~1.67) |
| [0.8, 1.5) | 1.12(1.08~1.17) | 1.14(1.03~1.26) | 1.02(0.96~1.08) |
| [1.5, 2.0) | 1.05(1.01~1.09) | 0.93(0.84~1.03) | 1.02(0.97~1.08) |
| [2.0, 2.5) | 1.00 (Ref.) | 1.00 (Ref.) | 1.00 (Ref.) |
| [2.5, 3.0) | 0.97(0.92~1.02) | 0.98(0.86~1.11) | 0.92(0.85~1) |
| [3.0, 4.0) | 1.01(0.95~1.08) | 1.08(0.92~1.27) | 1.18(1.07~1.3) |
| >=4.0 | 1.08(0.93~1.25) | 1.29(0.92~1.81) | 1.12(0.89~1.4) |
| Systemic immune-inflammation index (SII) |  |  |  |
| <300 | 1.07(1.01~1.13) | 1.12(0.97~1.3) | 1.12(1.03~1.21) |
| [300, 400) | 1.05(1~1.1) | 1.13(0.99~1.28) | 0.99(0.92~1.07) |
| [400, 500) | 1.00 (Ref.) | 1.00 (Ref.) | 1.00 (Ref.) |
| [500, 600) | 1.02(0.97~1.07) | 1.12(0.98~1.28) | 1.01(0.94~1.09) |
| [600, 800) | 1.06(1.01~1.11) | 1.13(1~1.28) | 1.05(0.98~1.12) |
| [800, 1500) | 1.12(1.07~1.18) | 1.39(1.23~1.57) | 1.06(0.99~1.14) |
| >=1500 | 1.5(1.37~1.64) | 2.37(1.93~2.91) | 1.28(1.11~1.48) |
| Neutrophil-to-lymphocyte ratio (NLR) |  |  |  |
| <1.5 | 1.00 (Ref.) | 1.00 (Ref.) | 1.00 (Ref.) |
| [1.5, 2.0) | 1(0.96~1.05) | 1(0.88~1.13) | 0.98(0.91~1.05) |
| [2.0, 2.5) | 1.05(1~1.11) | 1.11(0.98~1.25) | 1.01(0.94~1.09) |
| [2.5, 3.0) | 1.09(1.04~1.15) | 1.09(0.95~1.24) | 1.01(0.94~1.1) |
| [3.0, 3.5) | 1.15(1.08~1.22) | 1.23(1.05~1.43) | 1.1(1.01~1.2) |
| [3.5, 5.0) | 1.26(1.19~1.34) | 1.44(1.25~1.66) | 1.08(0.99~1.18) |
| >=5.0 | 1.45(1.34~1.58) | 1.88(1.55~2.28) | 1.21(1.07~1.38) |
| Platelet-to-lymphocyte ratio (PLR) |  |  |  |
| <80 | 1.07(1.02~1.14) | 1.15(1~1.33) | 1.12(1.03~1.21) |
| [80, 100) | 1.04(0.99~1.09) | 1.11(0.98~1.26) | 1.01(0.94~1.08) |
| [100, 120) | 1.00 (Ref.) | 1.00 (Ref.) | 1.00 (Ref.) |
| [120, 150) | 1.01(0.97~1.06) | 1.04(0.93~1.16) | 0.98(0.92~1.05) |
| [150, 200) | 1.05(1~1.1) | 1(0.89~1.13) | 0.97(0.91~1.04) |
| [200, 250) | 1.03(0.96~1.09) | 1.26(1.08~1.47) | 1.01(0.92~1.1) |
| >=250 | 1.19(1.1~1.29) | 1.44(1.19~1.74) | 1.12(0.99~1.26) |
| Lymphocyte-to-monocyte ratio (LMR) |  |  |  |
| <2.5 | 1.36(1.28~1.45) | 1.68(1.42~1.99) | 1.15(1.05~1.27) |
| [2.5, 3.0) | 1.17(1.1~1.25) | 1.58(1.33~1.87) | 0.98(0.89~1.08) |
| [3.0, 4.0) | 1.12(1.06~1.19) | 1.25(1.07~1.46) | 1.03(0.94~1.12) |
| [4.0, 5.0) | 1.05(0.99~1.12) | 1.23(1.05~1.44) | 0.97(0.89~1.06) |
| [5.0, 6.0) | 1.04(0.97~1.11) | 1.12(0.95~1.34) | 0.96(0.87~1.05) |
| [6.0, 8.0) | 1.00 (Ref.) | 1.00 (Ref.) | 1.00 (Ref.) |
| >=8.0 | 1.01(0.93~1.11) | 1.25(0.99~1.57) | 1.06(0.93~1.2) |
| Ref., Reference.  Adjusted age, sex, race, townsend deprivation index, education, BMI, smoking status, frequency of alcohol drinking, physical activity, hypertension, heart attack, angina, stroke, diabetes, hyperlipidemia, diseases of blood and blood-forming organs, chronic diseases involving the immune mechanism, and malignant neoplasms. | | | |

**Table S9.** The hazard risk (HR) with 95% confidence intervals (95%CI) between various systematic information indicators and three arrhythmia subtypes excluding participants with heart diseases, diseases of blood and blood-forming organs, chronic diseases involving the immune mechanism, or malignant neoplasms at baseline (N=137,477) by the Cox proportional hazard model.

| Variables | Atrial Fibrillation/Flutter | Ventricular Arrhythmia | Bradyarrhythmia |
| --- | --- | --- | --- |
|  | **HR (95%CI)** | **HR (95%CI)** | **HR (95%CI)** |
| C-reactive protein (mg/L) |  |  |  |
| <0.5 | 1.00 (Ref.) | 1.00 (Ref.) | 1.00 (Ref.) |
| [0.5, 1.0) | 0.99(0.93~1.06) | 1.05(0.89~1.25) | 1.04(0.95~1.15) |
| [1.0, 2.0) | 0.96(0.91~1.03) | 1.27(1.08~1.5) | 1(0.91~1.1) |
| [2.0, 3.0) | 1.01(0.94~1.08) | 1.12(0.92~1.36) | 1.05(0.95~1.17) |
| [3.0, 4.0) | 1.04(0.96~1.13) | 1.36(1.09~1.69) | 1.1(0.97~1.25) |
| [4.0, 10.0) | 1.09(1.02~1.18) | 1.39(1.14~1.69) | 1.14(1.02~1.28) |
| >=10.0 | 1.25(1.14~1.38) | 1.91(1.5~2.43) | 1.26(1.09~1.47) |
| Neutrophil count (10^9 cells/L) |  |  |  |
| <2.0 | 1.00 (Ref.) | 1.00 (Ref.) | 1.00 (Ref.) |
| [2.0, 3.0) | 1.1(0.93~1.29) | 1.02(0.68~1.55) | 0.84(0.68~1.05) |
| [3.0, 4.0) | 1.04(0.89~1.22) | 1.1(0.73~1.64) | 0.88(0.71~1.09) |
| [4.0, 5.0) | 1.08(0.92~1.27) | 1.03(0.69~1.54) | 0.9(0.72~1.11) |
| [5.0, 6.0) | 1.11(0.94~1.31) | 1.39(0.93~2.1) | 0.94(0.76~1.18) |
| [6.0, 7.5) | 1.22(1.03~1.44) | 1.41(0.92~2.15) | 0.95(0.75~1.19) |
| >=7.5 | 1.4(1.16~1.68) | 1.9(1.21~2.99) | 0.99(0.76~1.29) |
| Monocyte count (10^9 cells/L) |  |  |  |
| <0.3 | 1.00 (Ref.) | 1.00 (Ref.) | 1.00 (Ref.) |
| [0.3, 0.4) | 1.02(0.94~1.1) | 0.99(0.8~1.23) | 1.02(0.9~1.15) |
| [0.4, 0.5) | 1.05(0.97~1.13) | 1.02(0.83~1.26) | 1(0.89~1.13) |
| [0.5, 0.6) | 1.02(0.95~1.11) | 1.19(0.96~1.46) | 1.01(0.89~1.14) |
| [0.6, 0.7) | 1.08(0.99~1.17) | 1.13(0.9~1.42) | 1.03(0.9~1.16) |
| [0.7, 0.8) | 1.17(1.07~1.29) | 1.38(1.08~1.77) | 1.13(0.98~1.31) |
| >=0.8 | 1.19(1.08~1.31) | 1.67(1.3~2.13) | 1.04(0.89~1.2) |
| Lymphocyte count (10^9 cells/L) |  |  |  |
| <0.8 | 1.49(1.21~1.84) | 1.14(0.61~2.13) | 1.27(0.91~1.78) |
| [0.8, 1.5) | 1.12(1.06~1.17) | 1.18(1.03~1.35) | 1.07(0.98~1.15) |
| [1.5, 2.0) | 1.04(1~1.09) | 0.99(0.87~1.12) | 1.06(0.99~1.14) |
| [2.0, 2.5) | 1.00 (Ref.) | 1.00 (Ref.) | 1.00 (Ref.) |
| [2.5, 3.0) | 0.96(0.9~1.03) | 1.01(0.86~1.19) | 0.95(0.86~1.05) |
| [3.0, 4.0) | 0.95(0.87~1.04) | 1.06(0.86~1.32) | 1.16(1.02~1.31) |
| >=4.0 | 0.93(0.75~1.16) | 1.19(0.72~1.96) | 0.78(0.54~1.12) |
| Systemic immune-inflammation index (SII) |  |  |  |
| <300 | 1.06(0.99~1.13) | 1.28(1.07~1.54) | 1.1(0.99~1.22) |
| [300, 400) | 1.06(1~1.13) | 1.17(0.99~1.37) | 1.05(0.96~1.15) |
| [400, 500) | 1.00 (Ref.) | 1.00 (Ref.) | 1.00 (Ref.) |
| [500, 600) | 1.04(0.98~1.1) | 1.2(1.01~1.41) | 1.07(0.98~1.17) |
| [600, 800) | 1.04(0.98~1.1) | 1.11(0.95~1.31) | 1.09(1~1.19) |
| [800, 1500) | 1.14(1.07~1.21) | 1.6(1.37~1.87) | 1.15(1.05~1.26) |
| >=1500 | 1.49(1.31~1.7) | 2.56(1.91~3.43) | 1.22(0.98~1.51) |
| Neutrophil-to-lymphocyte ratio (NLR) |  |  |  |
| <1.5 | 1.00 (Ref.) | 1.00 (Ref.) | 1.00 (Ref.) |
| [1.5, 2.0) | 1(0.94~1.06) | 1.05(0.9~1.23) | 1.02(0.93~1.11) |
| [2.0, 2.5) | 1.06(1~1.12) | 1.06(0.9~1.24) | 1.05(0.96~1.15) |
| [2.5, 3.0) | 1.08(1.02~1.15) | 1.09(0.92~1.3) | 1.02(0.93~1.13) |
| [3.0, 3.5) | 1.15(1.07~1.24) | 1.21(1~1.47) | 1.13(1.01~1.26) |
| [3.5, 5.0) | 1.25(1.16~1.35) | 1.57(1.3~1.88) | 1.19(1.07~1.33) |
| >=5.0 | 1.48(1.32~1.65) | 1.82(1.38~2.39) | 1.23(1.03~1.47) |
| Platelet-to-lymphocyte ratio (PLR) |  |  |  |
| <80 | 1.08(1~1.15) | 1.18(0.98~1.42) | 1.16(1.04~1.29) |
| [80, 100) | 1.03(0.97~1.1) | 1.09(0.93~1.28) | 1.03(0.94~1.14) |
| [100, 120) | 1.00 (Ref.) | 1.00 (Ref.) | 1.00 (Ref.) |
| [120, 150) | 1.02(0.97~1.08) | 1.05(0.91~1.21) | 1.03(0.95~1.12) |
| [150, 200) | 1.05(0.99~1.11) | 1.09(0.94~1.27) | 1.06(0.98~1.15) |
| [200, 250) | 1.02(0.94~1.1) | 1.21(0.99~1.48) | 1.09(0.97~1.23) |
| >=250 | 1.16(1.05~1.29) | 1.28(0.97~1.68) | 1.13(0.96~1.33) |
| Lymphocyte-to-monocyte ratio (LMR) |  |  |  |
| <2.5 | 1.35(1.25~1.46) | 1.54(1.24~1.9) | 1.18(1.05~1.34) |
| [2.5, 3.0) | 1.24(1.15~1.34) | 1.54(1.25~1.89) | 1.07(0.95~1.21) |
| [3.0, 4.0) | 1.14(1.06~1.22) | 1.14(0.95~1.38) | 1.05(0.94~1.17) |
| [4.0, 5.0) | 1.07(0.99~1.15) | 1.09(0.9~1.32) | 0.99(0.89~1.1) |
| [5.0, 6.0) | 1.08(1~1.17) | 1.02(0.83~1.25) | 1.02(0.91~1.15) |
| [6.0, 8.0) | 1.00 (Ref.) | 1.00 (Ref.) | 1.00 (Ref.) |
| >=8.0 | 0.94(0.84~1.05) | 1.21(0.91~1.59) | 1.08(0.92~1.28) |
| Ref., Reference.  Adjusted age, sex, race, townsend deprivation index, education, BMI, smoking status, frequency of alcohol drinking, physical activity, hypertension, stroke, diabetes, and hyperlipidemia. | | | |
